# Mobile medication manager application to improve adherence with immunosuppressive therapy in renal transplant recipients: A randomized controlled trial

**DOI:** 10.1371/journal.pone.0224595

**Published:** 2019-11-05

**Authors:** Ahram Han, Sang-il Min, Sanghyun Ahn, Seung-Kee Min, Hye-jin Hong, Nayoung Han, Yon Su Kim, Curie Ahn, Jongwon Ha

**Affiliations:** 1 Department of Surgery, Seoul National University College of Medicine, Seoul, Korea; 2 Research Institute of Pharmaceutical Science, College of Pharmacy, Seoul National University, Seoul, Korea; 3 Department of Internal Medicine, Seoul National University College of Medicine, Seoul, Korea; 4 Transplantation Research Institute, Seoul National University College of Medicine, Seoul, Korea; Weill Cornell Medical College in Qatar, QATAR

## Abstract

**Background:**

Nonadherence to immunosuppressive therapy after renal transplantation is associated with poor graft outcomes. We aimed to evaluate whether the use of the Adhere4U mobile medication manager application could improve adherence among renal transplant recipients ≥1 year posttransplantation. Adhere4U can provide medication reminders, monitor medication use, and provide information on immunosuppressants.

**Methods:**

We conducted a prospective randomized controlled study to compare the rate of nonadherence to index immunosuppressant (tacrolimus or cyclosporine) in a group using the Adhere4U app (mobile group) and in another group receiving conventional care (control group). The primary outcome was the nonadherence rate, which was evaluated using an electronic medication event monitoring system during the 6-month intervention period. Our secondary outcome included self-reported adherence using the Basel Assessment of Adherence to Immunosuppressive Medication Scale (BAASIS) and the visual analog scale (VAS) based on a 4-week recall on days 28, 90, and 180. Longitudinal data of repeated measures of self-rated adherence were analyzed using generalized estimating equations (GEE) to compare the between-group difference in adherence change over time.

**Results:**

Between November 2013 and May 2015, 138 renal transplant recipients were randomly allocated to the control (n = 67) or the mobile group (n = 71). The overall nonadherence rate over the 6-month study period by electronic monitoring was 63.6%, with no between-group difference [mobile group, 65.0% (n = 39/60); control group, 62.1% (n = 36/58); odds ratio 1.14; 95% confidence interval 0.53–2.40; *p* = 0.89]. Self-rated nonadherence assessed using the BAASIS and VAS at baseline was 53.7% and 51.5%, respectively. Although the self-rated nonadherence by BAASIS of the mobile group was lower than the control group throughout the study period, there was no between-group difference in the change of nonadherence over time (χ2 = 2.82, df = 3, *p* = 0.42 by logistic GEE). There also was no significant between-group difference in the nonadherence by VAS (χ2 = 1.71, df = 3, *p* = 0.63 by logistic GEE) over time. The main limitation of this study was the low rate of patient engagement with the app among the mobile group. The rate of app use was 47.6% (31/65) at 28 days, 33.9% (19/56) at 90 days, and 11.5% (6/52) at 180 days.

**Conclusions:**

The Adhere4U application did not improve adherence to immunosuppressive therapy. Our evidence is limited by the high rate of attrition. Further studies on strategies to facilitate patient engagement with mobile interventions are warranted.

## Introduction

Lifelong immunosuppression is essential for successful renal transplantation. Nonadherence to immunosuppressive therapy (IST) is associated with poor outcomes including the development of de novo donor-specific antibodies [1], late acute rejection, graft failure [2], and mortality [3]. Nevertheless, nonadherence after renal transplantation is surprisingly prevalent, occurring in up to 65% of patients [4]. Promoting adherence has been challenging, with nonadherence being influenced by multiple factors including a lack of social support, dialysis experience, the complexity of the treatment regimen, forgetfulness, intentional nonadherence, a sense of autonomy, and beliefs regarding medication [5]. As the effects of these intentional and unintentional factors vary among individuals, interventions to improve adherence should be multidimensional and tailored [6,7].

Mobile health applications (apps) are emerging as tools that have the potential to address the different factors that influence nonadherence. These apps are easily accessible and can be tailored to meet the specific needs of a patient group, including real-time monitoring of medication use and prompting [8]. Although numerous generic medication management apps are available, the use of customized apps for specific patient groups is only beginning to be developed and tested [9–11]. Promising results have been reported from studies involving patients with hypertension [12], epilepsy [13], and HIV infection [14] using medication apps to enhance their medication adherence. Regarding renal transplantation, several qualitative studies were recently published that evaluated the perceived benefits of an app for improving adherence [15,16]. However, well-designed controlled trials assessing the efficacy of customized apps in renal transplant recipients (RTR) are currently lacking.

In this manuscript, we describe the Adhere4U app that we developed to promote adherence to IST among RTRs in Korea. The app provides medication reminders, tracks medication use trends, and provides information on immunosuppressants. We hypothesized that 6 months of Adhere4U app use would improve adherence in RTRs. The primary outcome of our trial was the nonadherence rate, which was calculated using the electronic monitoring system, and the secondary outcome was self-reported nonadherence.

## Materials and methods

We conducted the PRIMA (imPRoving adherence to Immunosuppressive therapy by Mobile internet Application) trial to test our hypothesis that 6 months of Adhere4U app use would improve adherence in RTRs. The trial was a prospective, single-center, parallel-group, and randomized controlled study, to compare the rate of nonadherence to index medication (tacrolimus or cyclosporine) in one group using the Adhere4U app (mobile group) and in another group receiving conventional care (control group). The study protocol was approved by the institutional review board of Seoul National University Hospital (IRB no. 1306-031-496, Clinicaltrials.gov: NCT 01905514).

### Study population

Outpatients who had received a renal allograft ≥1 year prior to the trial enrollment were recruited based on the following inclusion criteria: aged 15–70 years, Android smartphone user, and current use of twice-daily tacrolimus or cyclosporine as the principal immunosuppressant. The lower age limit was set to 15 years as this is the age at which Korean adolescents enter high school, where they spend more than 12 hours a day; it is therefore an age at which self-care is required in terms of medication-taking behavior. The patients with once or thrice daily tacrolimus were excluded because of a possible discrepancy in the baseline adherence rate [17,18] and the questionable equivalence of the pharmacologic effects of specific nonadherent behavior (e.g., single missed dose or delayed dose) among patients on different dosing regimens, considering their differing pharmacokinetic profiles [19]. Other exclusion criteria included multiorgan transplantation, change in IST regimen <4 weeks before enrollment, medication managed by a caregiver, inability to use a medication event monitoring system (MEMS) bottle, and women who were pregnant or planned to become pregnant within 6 months.

### Randomization and blinding

**Fig 1:**
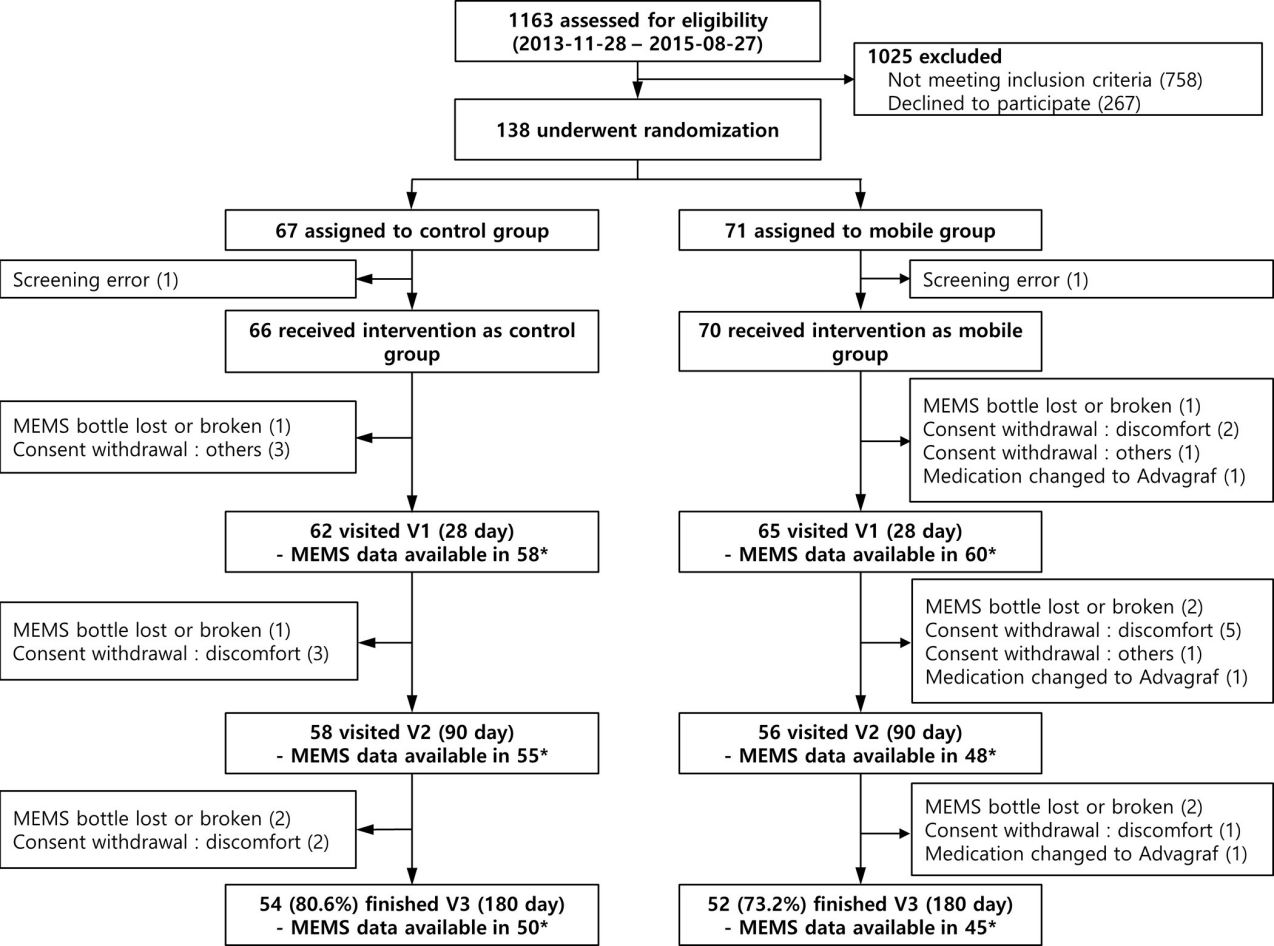
CONSORT diagram.

Fig 1 shows the flow diagram for the study. Participants were randomized 1:1 to the mobile or the control group. Group allocation was performed using a web-based scheme (permuted-block randomization with a concealed and varied block size) that was managed by the Medical Research Collaborating Centre (https://mrcc.snuh.org) of Seoul National University Hospital. Clinicians and survey assessors were blinded to the group assignment. Blinding of study participants and coordinators was impossible, as providing education on the app, as well as its use, was an essential component of the study.

### Study intervention

Adhere4U, a medication management smartphone app developed for transplantation patients in Korea was provided to the study patients enrolled in the intervention arm (https://play.google.com/store/apps/details?id=com.snuh.medinfo). The features of the app were as follows (S1 Fig): 1) audible and/or visual reminders able to reflect medication tapers, nondaily administration, and start and stop dates; 2) personal tracking data on missed and taken doses by providing a daily checklist of medication and when it was taken; 3) patient’s medication adherence report; 4) detailed information on all immunosuppressants; 5) an educational video on the importance of IST; and 6) patient’s laboratory test results. The medication adherence provided by the app was calculated by dividing the sum of alarms checked as taken divided by the sum of all scheduled alarms during the period. The medication reminders and data recording components of the app were designed to run without internet connectivity.

During the initial visit, the app was installed on the smartphone of patients in the mobile group, and education on its use was provided. The time log data including when prompting cues were provided, as well as when they were turned off, were automatically uploaded to the central database and were later used to evaluate patient engagement with the app. At enrollment, patients in both study groups were once again educated on the importance of adherence and were taught to take calcineurin inhibitors every 12 hours whilst avoiding food 2 hours before and 1 hour after taking their medication.

### Data collection

**Fig 2:**
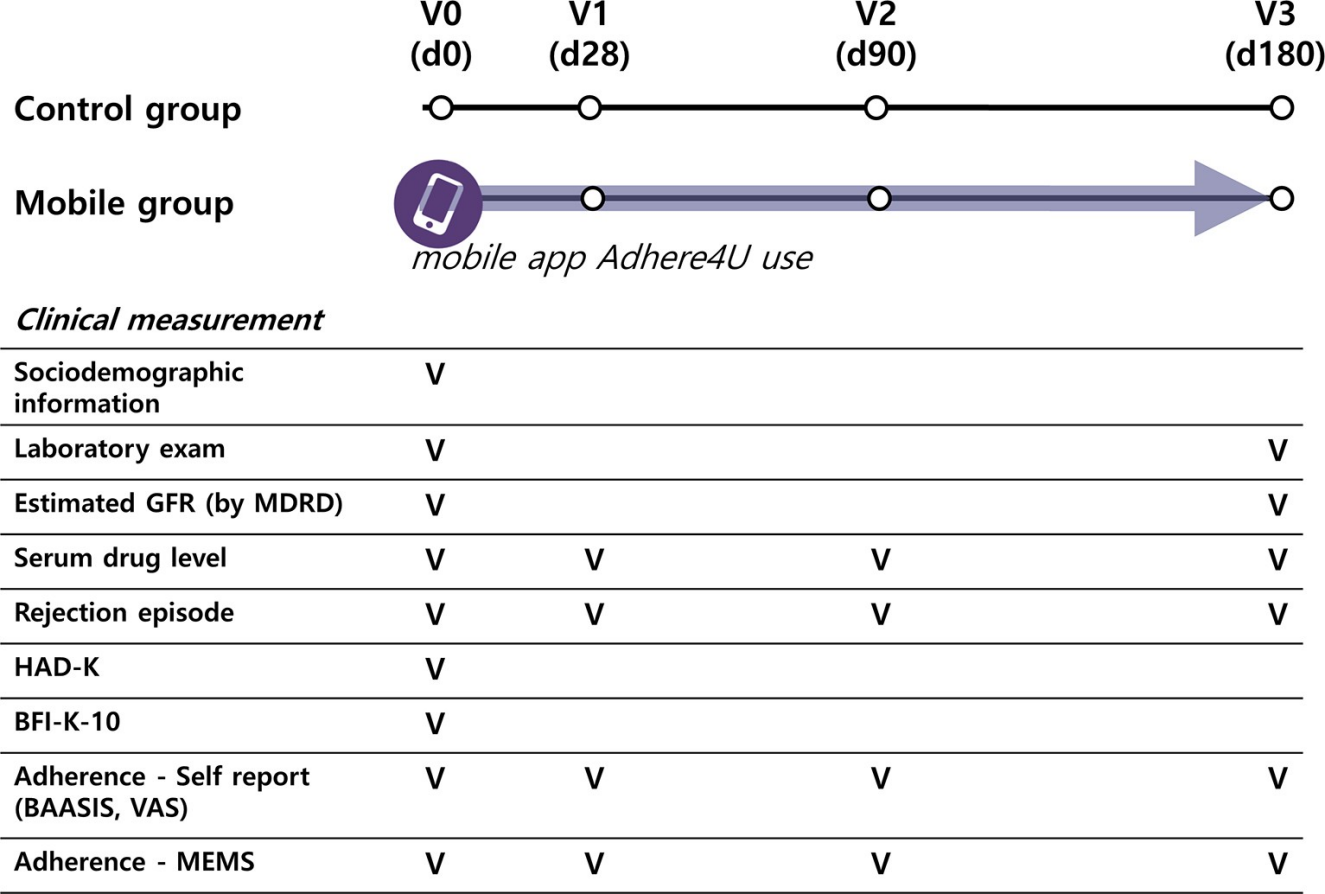
Study scheme.

Adherence was assessed using electronic monitoring (EM) and self-reports (Fig 2). The use of calcineurin inhibitor that was to be taken twice daily was selected as the target drug for EM. For EM, a medication bottle with MEMS V prescription container lids (AARDEX Ltd., Zug, Switzerland) was provided to both groups. The MEMS lids recorded the time and date of bottle openings onto a digital chip, and individual data were downloaded by the study pharmacists using a MEMS reader software program at each scheduled follow-up visit. Both the control group and the mobile group patients were asked to take the target drug from the MEMS bottle only.

Self-reported adherence was assessed using the Basel Assessment of Adherence to Immunosuppressive Medication Scale (BAASIS), as well as a visual analog scale (VAS) based on a 4-week recall on day 28, 90, and 180. The BAASIS included 4 items that assessed the taking and timing of medication use, drug holidays, and dose reduction on a 6-point scale, ranging from never (0) to every day (5) [20]. The VAS score ranged from 0 (never took the medication as prescribed) to 100 (always took the medication as prescribed). Nonadherence was defined as a positive answer to any of the 4 items (score ≥1) using the BAASIS [20], and as a score other than 100 using the VAS.

Sociodemographic information including anxiety, depression, and personality factors were recorded at baseline. Anxiety and depression were assessed using the Hospital Anxiety and Depression Scale (HADS) [21,22], and personality factors of the 10-item Big Five Inventory (BFI-10) [23,24]. Validated Korean versions of both the HADS [22] and the BFI-10 (BFI-10-K) [24] were administered to all patients at enrollment.

### Primary and secondary outcomes

The following adherence data were measured using the EM of medication—taking adherence (the proportion of prescribed doses taken), dosing adherence (the proportion of days on which the prescribed dose was taken), timing adherence (the proportion of between-dose intervals within 25% of the prescribed interval), and drug holidays (the number of periods without drug intake that was >3 times the prescribed interval) [25]. The primary outcome of our study was a binary indicator of the 6-month cumulative adherence based on the EM data. Nonadherence was defined as a taking adherence of <98% or >102% and/or at least one drug holiday. Our definition of nonadherence was a modification of that proposed by Schäfer-Keller et al. [25]; we added an upper limit of 102% for the taking adherence to include repeated overdose as nonadherent.

The secondary outcomes included the self-reported rate of nonadherence, individual EM proportions for each measurement interval, intra-individual variability of serum trough levels of the index medication [26], estimated MDRD glomerular filtration rate, and episodes of biopsy-proven acute rejection. Both groups’ dosing and timing adherence was also evaluated by evaluating the day-by-day proportion of patients with the correct dosing and timing. In addition, we evaluated the correlation between nonadherence measured by the various methods. We also sought to identify whether specific baseline sociodemographic factors (i.e. age, sex, education level, occupational status, and transplantation characteristics including donor type, time since transplant, reasons of end stage renal disease, dialysis history, type of immunosuppressant medications, and previous graft infection or rejection) were associated with various nonadherence measures (i.e., self-rated nonadherence and nonadherence by EM).

### Statistical analysis

For sample size calculation, the nonadherence rate of the control group was estimated to be 30%, based on a previous report of EM-detected nonadherence [4]. A sample size of 125 was set to achieve 80% statistical power to detect a 20% absolute decrease in the nonadherence rate in the mobile group, assuming a 2-sided hypothesis test with an α level of 0.05 [27]. As we had assumed a 10% dropout rate, we planned to enroll 69 participants in each group.

Between-group differences in dichotomous variables were compared using a chi-squared (χ^2^) test or Fisher’s exact test, and continuous variables using an independent sample *t*-test or a Mann-Whitney *U* test. Longitudinal data of repeated measures of self-rated adherence and daily binary indices of correct dosing and timing assessed by MEMS were analyzed using generalized estimating equations (GEE). With logistic GEE analysis, we tested whether the two groups differed in the change in self-rated nonadherence rate from baseline, to post-intervention (day 28, day 90, and day 180). We also tested whether the treatment effect was modified by time by including the interaction term (group x visit) in the model. The logistic GEE analysis was also performed to compare the binary outcome of daily timing and dosing by MEMS between the two groups across time (EM days). A multivariate logistic regression analysis was used to identify baseline risk factors of nonadherence. A correlation analysis (Spearman’s rho) was performed to evaluate the relationship between adherence rates measured by different assays. The primary outcome was calculated for patients whose EM data were available from at least one follow-up visit. All other analyses were performed using the modified intention to treat population, which excluded those who were erroneously screened and enrolled but excluded before the intervention.

All statistical tests were two-sided, and a *p*-value of <0.05 was considered significant. Statistical analyses were performed using SPSS (version 21.0; SPSS Inc., Chicago, IL) and R 3.3.2 (http://www.r-project.org).

## Results

Between November 28, 2013, and May 27, 2015, 138 RTRs were randomly assigned to the control group (*n* = 67) or the mobile group (*n* = 71) (Fig 1). A large number of RTRs were excluded after screening– 758 patients were ineligible, and 267 patients declined to participate. Main reasons for ineligibility were age (<15 y.o. or >70 y.o., *n* = 89), time since transplantation (< 1 year, *n* = 151), once daily or thrice daily tacrolimus (*n* = 125), multiorgan recipients (heart-kidney, *n* = 4; liver-kidney *n* = 10; simultaneous pancreas and kidney or pancreas after kidney, n = 42), recent change in IST regimen (*n* = 43), and use of cellular phones other than Android smartphones (*n* = 284). Among 138 RTRs enrolled, 2 patients were excluded before the intervention because of erroneous baseline screening (recent change to once daily tacrolimus, *n* = 1; and usage of smart phone with iOS, *n* = 1). The mobile group and the control group were well balanced in terms of demographic and clinical characteristics (Table 1).

**Table 1:** Baseline characteristics of the 136 patients who were randomized and received intervention as allocated.

	Total n = 136	Control group n = 66	Mobile group n = 70	*p*-value
***Sociodemographics***				
Age (years), median (IQR)	43 (34–53)	43 (30–52)	45 (35–54)	0.35
BMI, mean ± SD	22.2 ± 3.1	21.8 ± 2.7	22.5 ± 3.4	0.21
Male, n (%)	88 (64.7%)	45 (68.2%)	43 (61.4%)	0.52
Education level				0.48
Less than middle school	7 (5.1%)	5 (7.6%)	2 (2.9%)	
Middle school	19 (14.0%)	8 (12.1%)	11 (15.7%)	
High school	48 (35.3%)	21 (31.8%)	27 (38.6%)	
University	62 (45.6%)	32 (48.5%)	30 (42.9%)	
Occupation				0.81
Full time	67 (49.3%)	30 (45.5%)	37 (52.9%)	
Part time	9 (6.6%)	5 (7.6%)	4 (5.7%)	
Student	13 (9.6%)	8 (12.1%)	5 (7.1%)	
Housewife	26 (19.1%)	12 (18.2%)	14 (20.0%)	
Unemployed	21 (15.4%)	11 (16.7%)	10 (14.3%)	
Smoking				0.35
Current smoker	4 (2.9%)	3 (4.5%)	1 (1.4%)	
Previous smoker	1 (0.7%)	0 (0.0%)	1 (1.4%)	
Non smoker	131 (96.3%)	63 (95.5%)	68 (97.1%)	
***Clinical characteristics***				
Causes of ESRD				0.74
IgA nephropathy	30 (22.1%)	17 (25.8%)	13 (18.6%)	
Glomerulonephritis	16 (11.8%)	8 (12.1%)	8 (11.4%)	
ADPKD	15 (11.0%)	6 (9.1%)	9 (12.9%)	
Hypertension	8 (5.9%)	3 (4.5%)	5 (7.1%)	
Diabetes	8 (5.9%)	4 (6.1%)	4 (5.7%)	
FSGS	8 (5.9%)	3 (4.5%)	5 (7.1%)	
Vesicoureteral reflux	6 (4.4%)	3 (4.5%)	3 (4.3%)	
SLE	5 (3.7%)	4 (6.1%)	1 (1.4%)	
HSN	4 (2.9%)	2 (3.0%)	2 (2.9%)	
unknown	29 (21.3%)	11 (16.7%)	18 (25.7%)	
others	7 (5.1%)	5 (7.6%)	2 (2.9%)	
Dialysis before transplantation	114 (83.8%)	58 (87.9%)	56 (80.0%)	0.31
Dialysis duration, median months (IQR)	28.5 (3.0–67.3)	27.8 (6.0–62.7)	29.0 (2.8–72.0)	0.84
Months since transplantation, median (IQR)	24.6 (13.6–53.2)	21.3 (13.2–52.2)	27.2(14.2–57.4)	0.23
Donor type				0.87
Living donor				
- 1^st^ degree related	28 (20.6%)	14 (21.2%)	14 (20.0%)	
- other related	30 (22.1%)	14 (21.2%)	16 (22.9%)	
- spouse	20 (14.7%)	9 (13.6%)	15.7%)	
- other nonrelated	1 (0.7%)	1 (1.5%)	0 (0.0%)	
Deceased donor	57 (41.9%)	28 (42.4%)	29 (41.4%)	
Number of transplantation				0.65
First	128 (94.1%)	61 (92.4%)	67 (95.7%)	
Second	8 (5.9%)	5 (7.6%)	3 (4.3%)	
Number of IS				0.31
2	23 (16.9%)	14 (21.2%)	9 (12.9%)	
3	113 (83.1%)	52 (78.8%)	61 (87.1%)	
Type of calcineurin inhibitor				0.31
Cyclosporine A	8 (5.9%)	2 (3.0%)	6 (8.6%)	
Tacrolimus	128 (94.1%)	64 (97.0%)	64 (91.4%)	
Number of medication other than IS, median (IQR)	3.0 (2.0–5.0)	3.0 (2.0–4.0)	3.5 (2.0–5.0)	0.55
Previous acute rejection				0.32
None	94 (69.6%)	43 (65.2%)	51 (73.9%)	
1	31 (23.0%)	19 (28.8%)	12 (17.4%)	
2	8 (5.9%)	3 (4.5%)	5 (7.2%)	
≥ 3	2 (1.4%)	1 (1.5%)	1 (1.4%)	
Serious infection after transplantation	25 (18.4%)	14 (21.2%)	11 (15.7%)	0.55
Systolic blood pressure, mean ± SD	122.6 ± 10.2	121.2 ± 8.9	123.9 ± 11.2	0.13
Serum creatinine, mean ± SD	1.3 ± 0.3	1.3 ± 0.3	1.2 ± 0.3	0.26
MDRD GFR, median (IQR)	63.0 (53.4–74.0)	60.0 (53.3–73.9)	64.4 (55.5–74.3	0.36
6 mo. Intraindividual variability of CNI, median (IQR)	13.0 (8.6–18.8)	14.5 (8.6–21.4)	11.6 (8.3–17.7)	0.28
HADS anxiety score, median (IQR)	5 (3–7)	5 (3–7)	5 (3–7)	0.79
HADS depression score, median (IQR)	6 (4–8)	6 (3–8)	5.5 (4–8)	0.76
BFI-10 neuroticism score, median (IQR)	3.0 (2.0–3.5)	3.0 (2.0–3.5)	2.8 (2.0–3.5)	0.85
BFI-10 openness score, median (IQR)	3.5 (3.0–4.5)	3.5 (2.5–4.0)	3.5 (3.0–4.0)	0.97
BFI-10 extraversion score, median (IQR)	3.0 (2.5–3.3)	3.0 (2.8–3.3)	3.0 (2.5–3.3)	0.63
BFI-10 agreeableness score, median (IQR)	3.5 (3.0–4.0)	3.5 (3.0–4.0)	3.5 (3.0–4.0)	0.53
BFI-10 conscientiousness score, median (IQR)	3.5 (3.0–4.0)	3.5 (3.0–4.5)	3.5 (3.0–4.0)	0.75
Non-adherent (BAASIS)	73 (53.7%)	37 (56.1%)	36 (51.4%)	0.71
Non-adherent (VAS)	70 (51.5%)	38 (57.6%)	32 (45.7%)	0.23

SD, standard deviation; BMI, body mass index; ESRD, end stage renal disease; IgA, immunoglobulin A; ADPKD, autosomal dominant polycystic kidney disease; FSGS, focal segmental glomerulosclerosis; SLE, systemic lupus erythematosus; HSN, Henoch Schönlein nephritis; IS, immunosuppressant; IQR, interquartile range; MDRD GFR, glomerular filtration rate by Modification in Diet in Renal Disease study equation; CNI, calcineurin inhibitor; HADS, Hospital Anxiety and Depression Scale; BFI-10, 10-item Big Five Inventory; BAASIS, Basel Assessment of Adherence to Immunosuppressive Medication Scale; VAS, Visual Analog Scale.

The 6-month intervention was completed by 54 (80.6%) patients in the control group and 52 (73.2%) patients in the mobile group (Fig 1). Dropouts were mainly because of issues related to the MEMS bottle (loss or breakage, *n* = 9; discomfort with use, *n* = 13). The baseline characteristics of the patients who were excluded from the primary outcome analysis did not differ from those included except for the number of total medications taken other than immunosuppressants (S1 Table). There were no between-group difference in the baseline characteristics among the included patients (S2 Table).

Self-rated nonadherence assessed using the BAASIS and VAS at baseline was 53.7% and 51.5%, respectively (Table 1). The prevalence of self-rated non-adherence was similar for the control and mobile groups: BAASIS, 37/66 (control group, 56.1%) versus 36/70 (mobile group, 51.4%), *p* = 0.71; VAS, 38/66 (control group, 57.6%) versus 32/70 (mobile group, 45.7%), *p* = 0.23. Clinical factors associated with non-adherence on the BAASIS (S3 Table) included: donor type [living donor other than first degree relative or spouse versus first degree relative or spouse, odd ratio (OR) 5.26; deceased donor versus first degree relative or spouse, OR 2.97], time since transplantation (≥2 years versus <2 years, OR 3.07), and conscientiousness score on the BFI-10-K (OR 0.57). Clinical factors associated with nonadherence on the VAS (S4 Table) included sex (male versus female, OR 2.3) and conscientiousness score (OR 0.45). Although anxiety and depression were significantly associated with self-rated nonadherence in univariate analysis, the association was not significant in multivariate analysis (S3 and S4 Tables).

### Adherence measured by electronic monitoring

The overall nonadherence rate was 63.6% for the 118 patients with available EM data, with no between-group difference over the 6-month study period: 39/60 (65.0%) for the mobile group and 36/58 (62.1%) for the control group [Absolute risk reduction -2.9%, OR 1.14, 95% Confidence Interval (CI) 0.54–2.40, *p* = 0.89] (Table 2). The median medication-taking adherence, dosing adherence, and timing adherence were 96.7%, 92.0%, and 92.3%, respectively, for the control group and 95.1%, 85.7%, and 85.3%, for the mobile group. Due to a high dropout rate, patients' dosing and timing adherence by MEMS was also analyzed by evaluating the day-by-day proportion of patients with the correct dosing and timing, and between-group differences were evaluated using the GEE model. In both groups, there was a general decreasing trend in the daily proportions of patients with correct dosing and timing (S2 Fig). When logistic GEE analysis was performed, group (i.e. the app use) was not a significant variable for either correct dosing (χ2 = 0.41, df = 2, *p* = 0.52) or correct timing (χ2 = 0.86, df = 1, *p* = 0.77; S5 Table).

**Table 2:** Overall adherence by electronic monitoring.

	Total (*n* = 118)	Control group (*n* = 58)	Mobile group (*n* = 60)	Effect estimate (η^2^ or OR (95% CI))	*p*-value
Taking adherence (%), median (IQR)	96.5 (76.7–99.5)	96.7 (77.5–99.4)	95.1 (71.3–99.6)	0.0013	0.70
Dosing adherence (%), median (IQR)	89.3 (63.5–96.4)	92.0 (65.3–97.3)	85.7 (59.2–96.2)	0.0085	0.32
Timing adherence (%), median (IQR)	89.9 (59.6–98.0)	92.3 (61.3–98.0)	85.3 (55.4–97.5)	0.0056	0.42
Drug holiday (day), median (IQR)	1 (0–7)	1 (0–6)	0.5 (0–7.5)	0.0012	0.71
Overall nonadherence based on electronic monitoring					
Non-adherent, n (%)	75 (63.6)	36 (62.1)	39 (65.0)	1.14 (0.54–2.40)	0.89
Adherent, n (%)	43 (36.4)	22 (37.9)	21 (35.0)		

Odds ratios for outcomes of the mobile group are presented in reference to the control group. Effect estimate is presented with η^2^ ($=\frac{Z^{2}}{\sqrt{n}}$) for the Mann–Whitney U test and the odds ratio with a 95% confidence interval for the chi-squared (χ^2^) test or Fisher’s exact test. OR, odd ratio; CI, confidence interval; IQR, interquartile range.

### Adherence measured by self-reports

**Fig 3:**
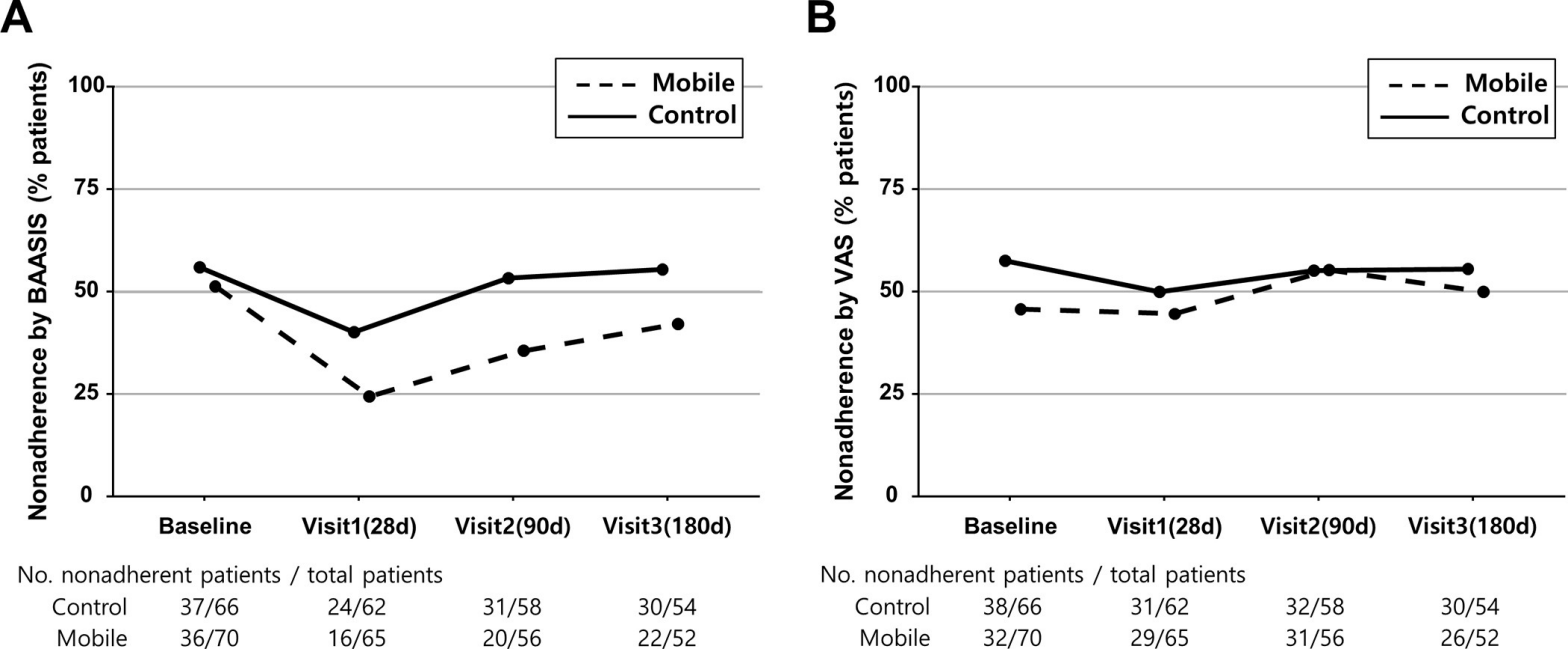
Self-rated Adherence Using the Basel Assessment of Adherence to Immunosuppressive Medication Scale (A) or the Visual Analog Scale (B).

The self-reported nonadherence rate over the study period is shown in Fig 3. Nonadherence rates by BAASIS were lower for the mobile group than the control group: 24.6% versus 38.7%, respectively, at day 28; 35.7% versus 53.4% at day 90; and 42.3% versus 55.5% at day 180. However, according to the logistic GEE model, there was no significant between-group difference in the change in the BAASIS nonadherence rate over time (χ2 = 2.82, df = 3, *p* = 0.42). Time was the only significant factor in the model, and the nonadherence rate at 28 days was significantly higher than that of the baseline (OR 0.54, 95% CI 0.35–0.84, *p* = 0.006). Time and the study group were not significantly associated with nonadherence according to the VAS (χ^2^ = 1.58, df = 1, *p* = 0.47 for study group; χ^2^ = 3.13, df = 3, *p* = 0.37 for time), and change in nonadherence rate over time was also not associated with the study group (χ2 = 1.71, df = 3, *p* = 0.63). Other secondary outcomes including the number of acute rejection episodes, change in estimated MDRD glomerular filtration rate, and intra-individual variability of serum trough levels of the index medication did not differ between the two groups (S6 Table).

### Engagement rate with the mobile app

**Fig 4:**
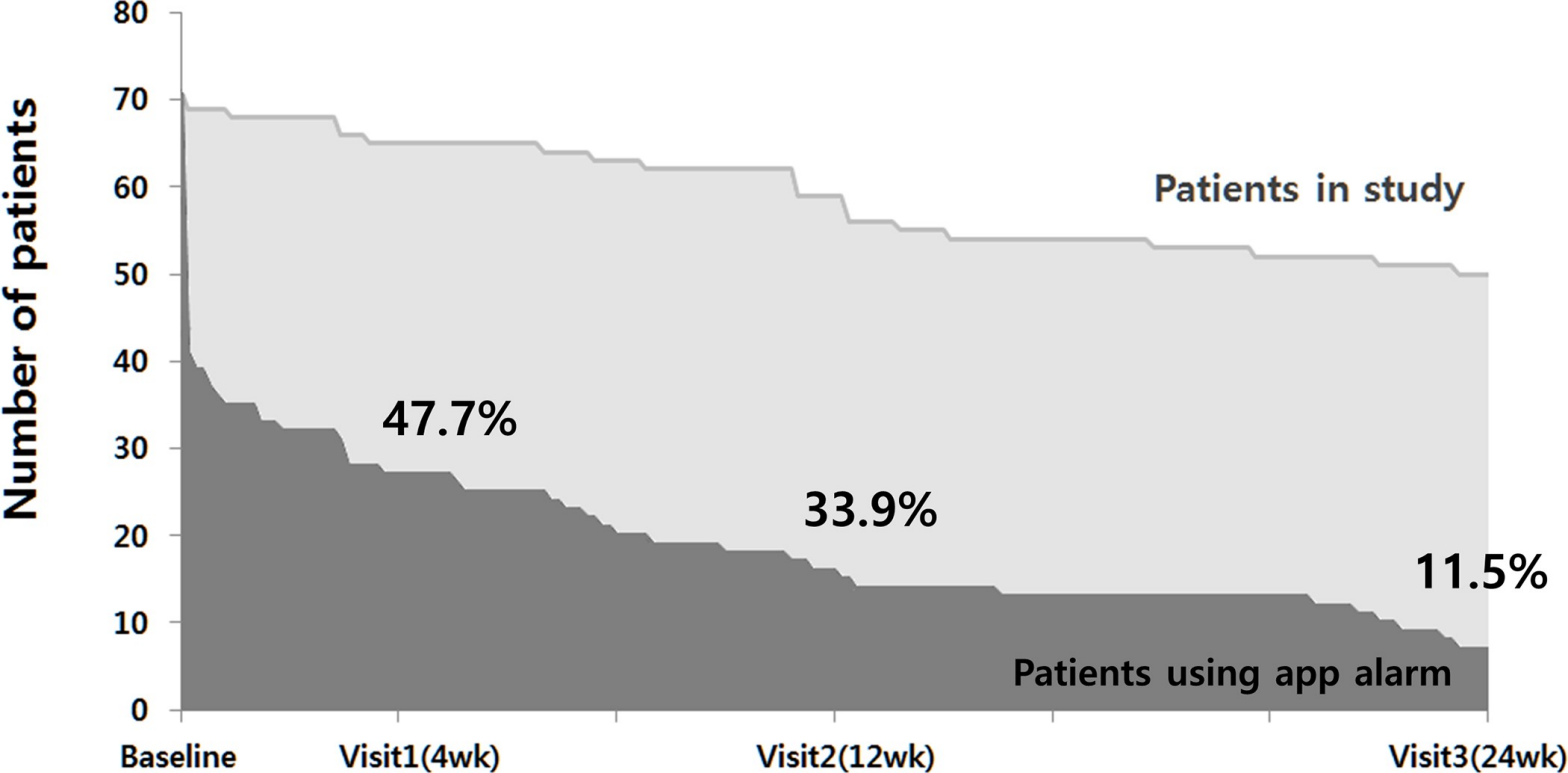
Application usage rate for the mobile group (patients using the app alarm/patients in the study).

Overall, app usage rate based on the use of the medication reminder function in the mobile group was low (Fig 4), with a rate of 47.6% at visit 1 (28 days), 33.9% at visit 2 (90 days), and 11.5% at visit 3 (180 days). Subgroup analysis of adherence between patients who discontinued using the app within 28 days and those who continued with its use ≥28 days within the mobile group showed increased taking, dosing and timing adherence by EM during the first month for those who continued using the app beyond 28 days (S7 Table). Adherence by the EM algorithm and self-rated adherence were also higher for this group but did not reach significance. Additional analysis was performed to identify patient factors associated with app engagement. However, no predictive factors were identified (S8 Table).

### Correlation between different measurement methods of nonadherence

The longitudinal adherence rate measured using EM, and the two self-reported measures were compared (Table 3). Self-rated adherence using the BAASIS and VAS was significantly correlated with the EM data (taking, dosing, and timing adherence) and the BAASIS and VAS were related to one another. Adherence data derived from the log data of the medication reminder was significantly associated with self-rated adherence and EM-dosing and EM-timing adherence, but not with the medication-taking adherence by EM.

**Table 3:** Spearman’s Rho correlation table for the different adherence measurement methods.

	EM algorithm	BAASIS	VAS	EM_taking	EM_dosing	EM_timing	VAS (scale)	App adherence
EM algorithm	1.000	0.231**	0.239**	0.772**	0.813**	0.781**	0.293**	0.098
BAASIS (0/1)		1.000	0.531**	0.202**	0.260**	0.273**	0.559**	0.312**
VAS (0/1)			1.000	0.154**	0.201**	0.215**	0.915**	0.207**
EM_taking				1.000	0.876**	0.895**	0.220**	0.154
EM_dosing					1.000	0.935**	0.260**	0.214**
EM_timing						1.000	0.298**	0.212**
VAS (scale)							1.000	0.239**
App adherence								1.00

EM, electronic monitoring; BAASIS, Basel assessment of adherence to immunosuppressive medications scale; VAS, visual analog scale; EM_taking, taking adherence by electronic monitoring; EM_dosing, dosing adherence by electronic monitoring; EM_timing, timing adherence by electronic monitoring.

*Correlation significant at the 0.05 level (2-tailed)

**Correlation significant at the 0.01 level (2-tailed)

## Discussion

To our knowledge, this study was among the first randomized controlled trials to present data on the feasibility and clinical efficacy of using a smartphone app to promote medication adherence among RTRs. Our motivation was to provide behavioral and educational intervention to improve adherence. With this aim, the app mainly targeted unintentional nonadherence [5,28,29] using reminders to re-establish medication routines and tracked medication taking to promote self-management. However, the 6-month intervention involving the mobile app failed to show a significant benefit regarding the overall 6-month cumulative nonadherence, as measured by EM, and the self-reported nonadherence that was assessed at 28, 90, and 180 days using the VAS and BAASIS. Our results were unexpected as automated reminders or text messages were previously reported to be useful for improving medication nonadherence in non-transplant [30,31] and transplant [32] settings.

The low rate of patient engagement with the app may partly explain the inferior outcomes with our app, as compared with simple text messages. One-half of the patients stopped using these reminders within the first month, and only 10% of the remaining patients were using the app up to the end of the study. Our findings are comparable to those reported for the “Medication Plan” app, for which only 29% of users who downloaded the app had regularly used it for >1 month [33]. This problem with attrition is a well-known feature of web-based interventions and is increasingly being recognized for mobile apps [34]. In line with the factors proposed by Eysenbach [34], several factors likely contributed to the high attrition rate, including the following issues. First, the prescheduled medication reminder of our app could be easily deleted with a single touch. Second, although we tracked personal medication data, this information was not used by clinicians to provide feedback (weak push factor). Lastly, it is necessary to consider that the study participants had undergone a renal transplant ≥1 year before this study and, therefore, were likely to have had an established medication routine with cues of their own (phone alarms or other available medication apps). The necessity to learn a new medication assistance program may not have been substantial compared to new RTRs.

The importance of patient engagement in mobile health intervention was also shown in a long-term follow-up study [35] of a recent randomized controlled trial evaluating the PocketPATH mobile app, which was designed to promote self-management for lung transplantation patients [36]. Despite a significant increase in short-term self-management behavior in the original study, there was no significant difference in most nonadherence elements in the follow-up study [35]. The authors speculated that the lack of sustained effect is likely to be related to a high rate of discontinuation of app use [35].

Our comparison of nonadherence between patients who stopped using the reminder function of the app at 28 days and those who continued its use for ≥28 days supports our hypothesis that a high attrition rate might have lowered the overall size of the effect of our intervention. Those who engaged with the app for ≥28 days had significantly higher medication-taking adherence, dosing adherence, and timing adherence with EM during the first month than those who were not engaged. Although this subgroup analysis was exploratory and thus needs validation, the results suggest that patient engagement may be the key in conducting a mobile intervention. Thus, how can we promote user engagement? Reported benefits of gaming and persuasive technology should be considered, including personalization, rewards, peer pressure, and clinician feedback that were found to be effective for web-based interventions [37,38]. Preselecting patients who are more likely to adhere to app interventions would be another strategy. Although we could not identify any predictive factors of continuous app engagement in this study, app-interventions may be more effective in newly transplanted RTRs as their motivation and need for support while adapting to a new life with IST is greater.

Another explanation for our negative result would be that intentional nonadherence may have been more prevalent than unintentional nonadherence in our study group. We designed our app mainly to target unintentional nonadherence based on previous evidence that nonadherence to IST among RTRs was mainly unintentional [28,29], with forgetfulness and interruption of daily routine identified as leading factors [39]. However, because whether nonadherence is intentional or unintentional can only be assessed by self-reporting, such an assessment is inherently biased by the desire to provide socially desirable answers [40]. The Adhere4U intervention would not have produced the anticipated effect on the overall nonadherence rate if intentional nonadherence was more prevalent than we had considered. This possibility points toward the need for studies exploring the effectiveness of interventions that target intentional nonadherence.

A few other features that were identified in our study are noteworthy. The overall rate of nonadherence was higher than the other reported rates measured with EM [4]. This might be explained in part by our strict definition of nonadherence, although this definition was also used in the SMART study [25]. Our higher nonadherence rate than that reported in the SMART study might be because of differences in patient characteristics: the SMART study included patients with longer working graft compared to our study (median 7 years versus 1.8 years, respectively). As such, a higher proportion of patients with good medication adherence might have been included in the SMART study.

We identified a low level of conscientiousness as a risk factor of self-reported nonadherence. In the only other study that considered the association between personality traits and nonadherence among RTRs, a lower openness score, but not low conscientiousness, was associated with nonadherence [41]. However, low conscientiousness was identified as a risk factor of nonadherence for heart, liver, and lung transplant recipients [42], as well as for patients with other chronic diseases [43]. The main aspects of conscientiousness—being organized, careful, self-regulatory and preferring planned behavior—may indeed help one build medication-taking habits and adhere to them. From a clinical view, the relationship of conscientiousness and adherence suggests a potential value of identifying patients with a low level of conscientiousness and providing targeted interventions that promote conscientiousness-related behaviors such as planning and impulse-control to improve adherence.

Prospective nonadherence data from different collection methods provided us an opportunity to compare various methods. We found a significant correlation between self-reported adherence and the rate measured using the EM algorithm, as well as EM taking, dosing, and timing adherence (*P* < 0.01). Our findings partly agree with those of Schafer-Keller [25], who reported a significant correlation between self-rated adherence with some of the adherence indices measured by MEMS (i.e. adherence by EM algorithm and timing adherence) but not with others (i.e. EM dosing and taking adherence). These differences may be explained by the different self-reporting methods used. We used the BAASIS, which considers 4 dimensions of medication adherence (taking, timing, drug holiday, and drug reduction) that may correlate more closely with adherence measured using EM than the Siegal scale that was used by Schafer-Keller. Of note, the adherence rate that was computed by the app was significantly correlated with EM dosing and timing adherence.

Our study had some limitations that necessitate acknowledgment. First, because of the nature of app use, blinding was not possible. Additionally, only patients from a single center were included. It is also important to note that Korea has the highest rate of smartphone use in the world (90%), with users installing, on average, 53 apps [44]. Therefore, our app-related intervention might not be suitable for populations with a lower prevalence of smartphone use.

The large number of patients excluded during screening also limits the generalizability of our study. The main reasons for exclusion were tacrolimus regimens other than twice daily regimen (i.e., once daily or thrice daily), usage of 2G phone or smartphone with iOS, and being < 1-year posttransplant. Especially excluding patients < 1-year posttransplant may have led to survival selection bias, as patients who were highly nonadherent to medication may have already lost their graft within the first year after transplant. Our lower age limit was set to 15 years. We had included high school aged adolescents in this study because we considered that their medication-taking behaviors were less likely to be under parental guidance considering the long time that Korean high school students spend outside the home on average. However, the youngest two patients included in the study were 18 years old and, therefore, the results of this study are not applicable to adolescents. The current study is also limited by the high rate of patients who refused to participate. Those who did not choose to participate may be more nonadherent than those who chose to participate, being less interested about medication taking behavior or uncomfortable about their medication behavior being monitored. And this selection bias may have led to over-estimation of baseline medication adherence.

The sample size of the current study was calculated assuming a 20% difference in the primary outcome (i.e., binary indicator of the 6 month cumulative EM adherence based on taking and drug holidays). Hence, the difference in nonadherence by self-report may not have been significant due to a lack of power. To reach a statistically significant difference, the calculated sample size would have needed to be much larger. In addition, although we had planned for a 10% dropout rate, only 77% of the patients completed the intervention, with discomfort with the use of the MEMS system as the primary reason for dropouts. The prototype MEMS used in this study significantly limited the ability to perform a standard measure of the effects of the intervention on nonadherence because of its impact on medication-taking behavior, as well as inconvenience regarding data collection. Although the MEMS provides some valuable details on individual nonadherence behavior, validated self-reports may be a better choice for evaluating the interventional effect in a trial setting. Recently introduced second- and third-generation MEMS devices with features of real-time data transmission or those that document actual medication ingestion also deserve mention as a promising alternative [45,46]. Lastly, although we had designed our control group to represent RTRs receiving routine care, initial re-education about correct medication-taking behavior provided upon trial enrolment and the perception of being monitored may have altered their adherence.

In summary, we did not identify a significant benefit regarding the use of the Adhere4U app for improving the rate of nonadherence among RTRs. Our evidence is limited by the high rate of attrition. Despite our negative results, mobile apps remain an attractive tool to support medication adherence. Future studies are warranted to enhance patient engagement with such apps.

## Supporting information

S1 Protocol Study protocol. (DOCX)

S1 Fig Features of the medication management smartphone app, Adhere4U. (PPTX)

S2 Fig Comparison of daily percentage of patients with (A) correct dosing and (B) correct timing.
(PPTX)

S1 Table Baseline characteristics of the patients included and excluded in the analysis. (DOCX)

S2 Table Characteristics of the two study group population included in the analysis for primary end point. (DOCX)

S3 Table Clinical factors associated with baseline self-rated nonadherence by BAASIS. (DOCX)

S4 Table Clinical factors associated with baseline self-rated nonadherence by VAS. (DOCX)

S5 Table GEE analysis for the interventional effect on (A) dosing and (B) timing by MEMS.
(DOCX)

S6 Table Secondary outcomes. (DOCX)

S7 Table Adherence of patients according to their app usage. (DOCX)

S8 Table Baseline characteristics of the patients according to their app usage. (DOCX)

## References

[1] Wiebe C, Gibson IW, Blydt-Hansen TD, Pochinco D, Birk PE, Ho J, et al Rates and determinants of progression to graft failure in kidney allograft recipients with de novo donor-specific antibody. Am J Transplant. 2015;15: 2921–2930. 10.1111/ajt.13347 26096305

[2] Denhaerynck K, Dobbels F, Cleemput I, Desmyttere A, Schafer-Keller P, Schaub S, et al Prevalence, consequences, and determinants of nonadherence in adult renal transplant patients: a literature review. Transpl Int. 2005;18: 1121–1133. 10.1111/j.1432-2277.2005.00176.x 16162098

[3] Prihodova L, Nagyova I, Rosenberger J, Majernikova M, Roland R, Groothoff JW, et al Adherence in patients in the first year after kidney transplantation and its impact on graft loss and mortality: a cross-sectional and prospective study. J Adv Nurs. 2014;70: 2871–2883. 10.1111/jan.12447 24853863

[4] Nerini E, Bruno F, Citterio F, Schena FP. Nonadherence to immunosuppressive therapy in kidney transplant recipients: can technology help? J Nephrol. 2016;29: 627–636. 10.1007/s40620-016-0273-x 26885659

[5] Rebafka A. Medication adherence after renal transplantation-a review of the literature. J Ren Care. 2016;42: 239–256. 10.1111/jorc.12181 27629770

[6] Fine RN, Becker Y, De Geest S, Eisen H, Ettenger R, Evans R, et al Nonadherence consensus conference summary report. Am J Transplant. 2009;9: 35–41. 10.1111/j.1600-6143.2008.02495.x 19133930

[7] Low JK, Williams A, Manias E, Crawford K. Interventions to improve medication adherence in adult kidney transplant recipients: a systematic review. Nephrol Dial Transplant. 2015;30: 752–761. 10.1093/ndt/gfu204 24950938

[8] Dayer L, Heldenbrand S, Anderson P, Gubbins PO, Martin BC. Smartphone medication adherence apps: potential benefits to patients and providers. J Am Pharm Assoc. 2013;53: 172–181. 10.1331/JAPhA.2013.12202 PMC3919626 23571625

[9] Fishbein JN, Nisotel LE, MacDonald JJ, Pensak NA, Jacobs JM, Flanagan C, et al Mobile application to promote adherence to oral chemotherapy and symptom management: a protocol for design and development. JMIR Res Protoc. 2017;6: e62 10.2196/resprot.6198 28428158 PMC5418526

[10] Kreyenbuhl J, Record EJ, Himelhoch S, Charlotte M, Palmer-Bacon J, Dixon LB, et al Development and feasibility testing of a smartphone intervention to improve adherence to antipsychotic medications. Clin Schizophr Relat Psychoses. 2016; 10.3371/csrp.krre.070816 27454213 PMC5910284

[11] Lakshminarayana R, Wang D, Burn D, Chaudhuri KR, Cummins G, Galtrey C, et al Smartphone- and internet-assisted self-management and adherence tools to manage Parkinson's disease (SMART-PD): study protocol for a randomised controlled trial. Trials. 2014;15: 374 10.1186/1745-6215-15-374 25257518 PMC4283131

[12] Patel S, Jacobus-Kantor L, Marshall L, Ritchie C, Kaplinski M, Khurana PS, et al Mobilizing your medications: an automated medication reminder application for mobile phones and hypertension medication adherence in a high-risk urban population. J Diabetes Sci Technol. 2013;7: 630–639. 10.1177/193229681300700307 23759395 PMC3869130

[13] Ernst LL, Harden CL, Pennell PB, Llewellyn N, Lau C, Barnard S, et al Medication adherence in women with epilepsy who are planning pregnancy. Epilepsia. 2016;57: 2039–2044. 10.1111/epi.13586 27778312 PMC6374285

[14] Perera AI, Thomas MG, Moore JO, Faasse K, Petrie KJ. Effect of a smartphone application incorporating personalized health-related imagery on adherence to antiretroviral therapy: a randomized clinical trial. AIDS Patient Care STDS. 2014;28: 579–586. 10.1089/apc.2014.0156 25290556 PMC4216527

[15] Israni A, Dean C, Kasel B, Berndt L, Wildebush W, Wang CJ. Why do patients forget to take immunosuppression medications and miss appointments: can a mobile phone app help? JMIR Public Health Surveill. 2016;2: e15 10.2196/publichealth.5285 27227150 PMC4869221

[16] Browning RB, McGillicuddy JW, Treiber FA, Taber DJ. Kidney transplant recipients' attitudes about using mobile health technology for managing and monitoring medication therapy. J Am Pharm Assoc. 2016;56: 450–454. 10.1016/j.japh.2016.03.017 PMC4968877 27450140

[17] Dobbels F, Berben L, De Geest S, Drent G, Lennerling A, Whittaker C, et al The psychometric properties and practicability of self-report instruments to identify medication nonadherence in adult transplant patients: a systematic review. Transplantation. 2010;90: 205–219. 10.1097/TP.0b013e3181e346cd 20531073

[18] Weng FL, Israni AK, Joffe MM, Hoy T, Gaughan CA, Newman M, et al Race and electronically measured adherence to immunosuppressive medications after deceased donor renal transplantation. J Am Soc Nephrol. 2005;16: 1839–48. 10.1681/ASN.2004121059 15800121

[19] Kuypers DR, Peeters PC, Sennesael JJ, Kianda MN, Vrijens B, Kristanto P, et al Improved adherence to tacrolimus once-daily formulation in renal recipients: a randomized controlled trial using electronic monitoring. Transplantation. 2013;95: 333–40. 10.1097/TP.0b013e3182725532 23263559

[20] Barraclough KA, Isbel NM, Johnson DW, Campbell SB, Staatz CE. Once-versus twice-daily tacrolimus. Drugs. 2011;71: 1561–77. 10.2165/11593890-000000000-00000 21861541

[21] Zigmond AS, Snaith RP. The hospital anxiety and depression scale. Acta Psychiatr Scand. 1983;67: 361–370. 10.1111/j.1600-0447.1983.tb09716.x 6880820

[22] Oh SM, Min KJ, Park DB. A study on the standardization of the hospital anxiety and depression scale for Koreans: a comparison of normal, depressed and anxious groups. J Korean Neuropsychiatr Assoc. 1999;38: 289–296.

[23] Rammstedt B, John OP. Measuring personality in one minute or less: a 10-item short version of the big five inventory in English and German. J Res Pers. 2007;41: 203–212.

[24] Kim SY, Kim JM, Yoo JA, Bae KY, Kim SW, Yang SJ, et al Standardization and validation of big five inventory-Korean version(BFI-K) in elders. Korean J Biol Psychiatry. 2010;17: 15–25.

[25] Schäfer-Keller P, Steiger J, Bock A, Denhaerynck K, De Geest S. Diagnostic accuracy of measurement methods to assess non-adherence to immunosuppressive drugs in kidney transplant recipients. Am J Transplant. 2008;8: 616–626. 10.1111/j.1600-6143.2007.02127.x 18294158

[26] Min SI, Ha J, Kang HG, Ahn S, Park T, Park DD, et al Conversion of twice-daily tacrolimus to once-daily tacrolimus formulation in stable pediatric kidney transplant recipients: pharmacokinetics and efficacy. Am J Transplant. 2013;13(8): 2191–2197. 10.1111/ajt.12274 23734831

[27] Hulley SB, Cummings SR, Browner WS, Grady D, Newman TB. Designing clinical research: an epidemiologic approach 4th ed. Philadelphia, PA: Lippincott Williams & Wilkins; 2013 pp. 75.

[28] Griva K, Davenport A, Harrison M, Newman SP. Non-adherence to immunosuppressive medications in kidney transplantation: intent vs. forgetfulness and clinical markers of medication intake. Ann Behav Med. 2012;44: 85–93. 10.1007/s12160-012-9359-4 22454221

[29] Muduma G, Shupo F, Dam S, Hawken N, Aballéa S, Odeyemi I, et al Patient survey to identify reasons for non-adherence and elicitation of quality of life concepts associated with immunosuppressant therapy in kidney transplant recipients. Patient Prefer Adherence. 2016;10: 27–36. 10.2147/PPA.S96086 26834463 PMC4716768

[30] Vervloet M, Linn AJ, van Weert JC, de Bakker DH, Bouvy ML, van Dijk L. The effectiveness of interventions using electronic reminders to improve adherence to chronic medication: a systematic review of the literature. J Am Med Inform Assoc. 2012;19: 696–704. 10.1136/amiajnl-2011-000748 22534082 PMC3422829

[31] Anglada-Martinez H, Riu-Viladoms G, Martin-Conde M, Rovira-Illamola M, Sotoca-Momblona JM, Codina-Jane C. Does mHealth increase adherence to medication? Results of a systematic review. Int J Clin Pract. 2015;69: 9–32. 10.1111/ijcp.12582 25472682

[32] Reese PP, Bloom RD, Trofe-Clark J, Mussell A, Leidy D, Levsky S, et al Automated reminders and physician notification to promote immunosuppression adherence among kidney transplant recipients: a randomized trial. Am J Kidney Dis. 2017;69: 400–409. 10.1053/j.ajkd.2016.10.017 27940063

[33] Becker S, Kribben A, Meister S, Diamantidis CJ, Unger N, Mitchell A. User profiles of a smartphone application to support drug adherence—experiences from the iNephro project. PLoS One. 2013;8: e78547 10.1371/journal.pone.0078547 24194946 PMC3806829

[34] Eysenbach G. The law of attrition. J Med Internet Res. 2005;7: e11 10.2196/jmir.7.1.e11 15829473 PMC1550631

[35] Geramita EM, Dabbs AJD, DiMartini AF, Pilewski JM, Switzer GE, Posluszny DM, et al Impact of a mobile health intervention on long-term nonadherence after lung transplantation: follow-up after a randomized controlled trial. Transplantation. 2019. [Epub ahead of print] 10.1097/TP.0000000000002872 PMC7170004 31335759

[36] Rosenberger EM, DeVito Dabbs AJ, DiMartini AF, Landsittel DP, Pilewski JM, Dew MA. Long-term follow-up of a randomized controlled trial evaluating a mobile health intervention for self-management in lung transplant recipients. Am J Transplant. 2017;17(5): 1286–1293. 10.1111/ajt.14062 27664940 PMC5365382

[37] Kelders SM, Kok RN, Ossebaard HC, van Gemert-Pijnen JE. Persuasive system design does matter: a systematic review of adherence to web-based interventions. J Med Internet Res. 2012;14: e152 10.2196/jmir.2104 23151820 PMC3510730

[38] Looyestyn J, Kernot J, Boshoff K, Ryan J, Edney S, Maher C. Does gamification increase engagement with online programs? a systematic review. PLoS One. 2017;12: e0173403 10.1371/journal.pone.0173403 28362821 PMC5376078

[39] Schmid-Mohler G, Thut MP, Wuthrich RP, Denhaerynck K, De Geest S. Non-adherence to immunosuppressive medication in renal transplant recipients within the scope of the integrative model of behavioral prediction: a cross-sectional study. Clin Transplant. 2010;24: 213–222. 10.1111/j.1399-0012.2009.01056.x 19674014

[40] Adams AS, Soumerai SB, Lomas J, Ross-Degnan D. Evidence of self-report bias in assessing adherence to guidelines. Int J Qual Health Care. 1999;11: 187–192. 10.1093/intqhc/11.3.187 10435838

[41] Gorevski E, Succop P, Sachdeva J, Cavanaugh TM, Volek P, Heaton P, et al Is there an association between immunosuppressant therapy medication adherence and depression, quality of life, and personality traits in the kidney and liver transplant population? Patient Prefer Adherence. 2013;7: 301–307. 10.2147/PPA.S34945 23620661 PMC3630988

[42] Dobbels F, Vanhaecke J, Dupont L, Nevens F, Verleden G, Pirenne J, et al Pretransplant predictors of posttransplant adherence and clinical outcome: an evidence base for pretransplant psychosocial screening. Transplantation. 2009;87: 1497–1504. 10.1097/TP.0b013e3181a440ae 19461486

[43] Molloy GJ, O'Carroll RE, Ferguson E. Conscientiousness and medication adherence: a meta-analysis. Ann Behav Med. 2014;47: 92–101. 10.1007/s12160-013-9524-4 23783830

[44] Kakihara M. Mobile Apps in APAC: 2016 report; 2016 [cited 2017 June 28]. Database: thinkwithgoogle [Internet]. Available from: http://apac.thinkwithgoogle.com/intl/en/articles/mobile-apps-in-apac-2016-report.html

[45] Haberer J. Medication event monitoring systems In: Gellman MD, Turner JR, editors. Encyclopedia of behavioral medicine. New York, USA: Springer; 2013 pp. 1215–1219.

[46] Park LG, Howie-Esquivel J, Dracup K. Electronic measurement of medication adherence. West J Nurs Res. 2015;37(1): 28–49. 10.1177/0193945914524492 24577868

