# Supplementary material for: Mobile medication manager application to improve adherence with immunosuppressive therapy in renal transplant recipients: A randomized controlled trial
Source: PLoS One. 2019 Nov 5;14(11):e0224595. doi: 10.1371/journal.pone.0224595 (PMC6830819; doi:10.1371/journal.pone.0224595)
Supplement: S1 Protocol — (DOCX) [file pone.0224595.s001.docx]

Improving Adherence to Immunosuppressive Therapy using a

Mobile Internet Application in Solid Organ Transplant Patients

(PRIMA study)

Protocol No. PRIMA-13-003

Study Period
2013, IRB Approval – 2015, 2 Years post-IRB Approval

Prof. Jongwon Ha

Department of Surgery

Seoul National University College of Medicine

**Summary**

| Title | Improving Adherence to Immunosuppressive Therapy using a Mobile Internet Application in Solid Organ Transplant Patients  (PRIMA study) |
| --- | --- |
| Principal Investigator | Prof. Jongwon Ha  Dept. of Surgery, Seoul National University College of Medicine |

| Purpose | To develop a mobile internet application that assists with medication in immunosuppressive therapy to improve immunosuppressant adherence in organ transplant patients |
| --- | --- |
| Study Period | Date of IRB approval – 2 years post-IRB approval |
| Subjects | Renal transplant recipients |
| Methods | 1. Development of a mobile internet application that assists with medication. 2. A randomized clinical trial is carried out, where 138 post-transplantation patients currently taking immunosuppressants are divided into a control group (69 patients) and a test group (69 patients) who are requested to use the newly developed application.  3. Adherence to immunosuppressants is assessed using an electronic pillbox (MEMS®). |
| Expected Outcome | Through this study, a mobile internet application will be developed to improve patients’ adherence to medication. The use of the application is likely to minimize nonadherence, the most important cause of preventable transplanted organ loss after transplantation, while allowing long-term survival of the transplanted organ. |

**Clinical Study Protocol**

1. **Research title and phase**
   Improving Adherence to Immunosuppressive Therapy using a Mobile Internet Application in Solid Organ Transplant Patients (PRIMA study)

Phase: Clinical trial led by the principal investigator

1. **Research institution: name and address**

Dept. of Surgery, Seoul National University College of Medicine

101 Daehak-ro, Jongno-gu, Seoul

1. **Principal investigator and co-investigators: name and title**
2. **Principal investigator**

Jongwon Ha
Professor
101 Daehak-ro, Jongno-gu, Seoul, Seoul National University College of Medicine

Dept. of Suregry

1. **Co-investigators**

Sang-il Min
Clinical Assistant Professor
101 Daehak-ro, Jongno-gu, Seoul, Seoul National University College of Medicine

Dept. of Surgery

Seung-Kee Min
Associate Professor
101 Daehak-ro, Jongno-gu, Seoul, Seoul National University College of Medicine

Dept. of Suregry

Curie Ahn
Professor
101 Daehak-ro, Jongno-gu, Seoul, Seoul National University College of Medicine

Dept. of Nephrology

Yon Su Kim
Professor
101 Daehak-ro, Jongno-gu, Seoul, Seoul National University College of Medicine

Dept. of Nephrology

Jae-Seok Yang
Clinical Associate Professor
101 Daehak-ro, Jongno-gu, Seoul, Seoul National University College of Medicine

Transplantation Research Institute

Hee-Kyeong Kang
Clinical Associate Professor
101 Daehak-ro, Jongno-gu, Seoul, Seoul National University College of Medicine

Dept. of Pediatrics

Jeong-Mi Oh
Professor
101 Daehak-ro, Jongno-gu, Seoul, Seoul National University College of Pharmacy

Research Institute of Pharmaceutical Science

Chan-Joong Choi
Clinical Fellow

101 Daehak-ro, Jongno-gu, Seoul, Seoul National University College of Medicine

Dept. of Suregry

Ahram Han
Clinical Fellow

101 Daehak-ro, Jongno-gu, Seoul, Seoul National University College of Medicine

Dept. of Suregry

Nayoung Han
Graduate Student
101 Daehak-ro, Jongno-gu, Seoul, Seoul National University College of Pharmacy

Research Institute of Pharmaceutical Science

Byeong-Soon Doh
Clinical Research Nurse
101 Daehak-ro, Jongno-gu, Seoul, Seoul National University College of Medicine

Dept. of Suregry

1. **Sub-investigator**

Sang-il Min
Clinical Assistant Professor
101 Daehak-ro, Jongno-gu, Seoul, Seoul National University College of Medicine

Dept. of Suregry

1. **Client
   1) Client: name and address**

NA **2) Monitoring agent: name and title**

NA

1. **Institution providing the research fund: name and address**

Astellas Pharma Inc. in Korea

Location: F6, 401 Hakdong-ro (Geumha Bld. Cheongdam-dong) Gangnam-gu, Seoul
Contact no.: +82 (0)2 3448 0504

1. **Expected duration of the clinical trial**

Date of IRB approval – 3 years post-IRB approval

1. **Subjects**

Renal transplant recipients

1. **Background and purpose**

**1) Background**

Adherence to post-transplantation medication has a profound influence on the prognosis of the transplanted organ. In particular, irregular doses of immunosuppressants result in low suppression of immunity which in turn causes acute or chronic rejection and antibody-mediated rejection, thereby inducing chronic organ damage eventually leading to the loss of the transplanted organ. In a meta-analysis of patients with a chronic disorder, nonadherence resulted in a 24.8 % morbidity. In transplant patients, the average nonadherence rate was 22.6 cases per 100 person/year, but it could reach as high as 53% in renal transplant patients and adolescent patients. According to the joint meeting report of the American Society of Transplantation, the American Society of Transplant Surgeons, Health Services and Resource Administration, the American Society of Nephrology, the International Pediatric Transplant Association, UNOS and NATCO, adherence is defined as ‘the extent to which the patient’s behavior matches the agreed upon prescriber’s recommendations’ while in another study, it is defined as ‘the extent to which the patient follows the prescribed dose and interval of medication’. Although improving the adherence to immunosuppressants in transplant patients is the most effective way to lower the risk of preventable graft loss, only a few effective methods have been developed to improve medication adherence. At present, nine different types of mobile internet applications are available, all of which have been developed for patients in an English-speaking country and are therefore not suitable for use by Korean patients. The available applications are as follows:

HealthPrize (iPhone and Android; free)
Pillboxie (iPhone; free)
MediRemind (iPhone, Blackberry and Android; $0.99)
Dosecast (iPhone; free)
MotionPHR Health Record (iPhone and Android; $9.99)

Medsy (iPhone; $1.99)
Rxmind Me (iPhone; $1.99)
MedCoach (iPhone and Android; free)

**2) Purpose**
To develop a mobile internet application aimed at improving medication adherence of Korean patients.

1. **Selection criteria & sample size and rationale**
2. **Inclusion criteria**
   • Patients who received organ transplantation at Seoul National University Hospital and are currently in outpatient follow-up monitoring
   • Patients who are android smartphone users
   • Patients who received transplantation ≥1 year prior to the study
   • Patients who are currently taking a calcineurin inhibitor (Tacrolimus or Cyclosporine) as their principal immunosuppressant
   • Patients aged between 15 and 70
3. **Exclusion criteria**

• Patients who are unable to use MEMS®
• Patients who received multiple organ transplants
• Patients whose immunosuppressant was changed within four weeks of enrollment
• Patients whose medication regimen is under caregiver management
• Patients who are unable to use the application function of the smartphone
• Patients who are pregnant or are planning pregnancy

1. **Sample size and rationale**

The present study targets 138 patients who underwent organ transplantation at Seoul National University Hospital and are currently taking an immunosuppressant under outpatient follow-up monitoring. The control and test groups have 69 subjects each.

The lack of studies on medication adherence in transplant patients in Korea makes it difficult to verify the level of adherence in Korean patients. Most previous studies on adherence, in overseas transplant patients, targeted 30–60 subjects, who showed 40%–70% adherence before behavioral intervention and 80%–95% adherence after behavioral intervention (Bleser LD, transplant international 2009). However, in consideration of the differences between overseas national insurance systems and the system in Korea where insurance covers all post-transplantation immunosuppressants, the nonadherence rate was assumed to be 30 % in the control group and 10 % in the test group. Based on MRCC consultation, the significance level was set at α = 0.05 and type II error (β) was set at 0.20 so that the power of the test was 80 %, resulting in the need for an estimated 125 subjects (62 per group). Estimating a 10 % dropout rate, the sample size was determined to be 138 (69 per group).

1. **Methods**
2. **Detailed description**

1. A mobile internet application for improving medication adherence (proposed name, Adhere4U) will be developed, whose functions include:

① Notifications of the times to take the medication (alarm, etc.)
② Encouraging the patient to check the times to take medication
③ Checking the test results (period can be set)
④ Allowing the data to be shared with the physician given the patient’s consent
⑤ Privacy protection: Deletion of all data upon the loss of the phone, etc.
⑥ A little game function

- The application does not have any functions that require the patient to provide personal information such as a request for membership, etc.
2. The subjects will be 138 patients (69 in both the control and test groups) who received organ transplantation at Seoul National University Hospital, are in outpatient follow-up monitoring, and who have been selected based on the selection criteria.
3. All patients will be requested to perform a self-reported assessment of adherence to immunosuppressant and comedication on day 0, 28, 90, and 180 using the Basel Assessment of Adherence with Immunosuppressive medication Scale (BAASIS) and the Visual Analog Scale (VAS). The adherence to immunosuppressant will be assessed by drug dose (tacrolimus or cyclosporine trough level) on day 0, 28, 90, and 180 using the medication event monitoring system (MEMS®). Also, to identify the psychosocial characteristics among the baseline characteristics, Hospital Anxiety and Depression Inventory (14 item, Oh et al.) and Personality Traits (BFI-K-10, Kim et al.) will be assessed.

4. The subjects in the control group will be requested to only use MEMS®, while those in the test group will be asked to use MEMS® and the newly developed mobile internet application simultaneously.

5. The allocation of subjects to the control and the test groups will use the web-based randomization of MRCC.

6. A longitudinal assessment of the improvement in adherence will be carried out over the 6-month period following randomization.

.

1. **Method of selecting the control and randomization**

The control of this clinical study consists of patients who are requested to only use MEMS®.

• Group 1: Control group; patients requested to only use MEMS®
• Group 2: Test group; patients requested to use MEMS® and the mobile internet application adhere4U simultaneously

For random allocation of subjects, the web-based program (<http://mrcc.snu.ac.kr>) of MRCC will be used for 1:1 randomization of the control and the test groups.

1. **Methods of monitoring and the monitored items and clinical test items**

| **Visit Schedule & Assessed Items** | | | | |
| --- | --- | --- | --- | --- |
| **Item** | **Screening**  **(Day 0)** | **Day 28** | **Day 90** | **Day 180** |
| **Informed Consent Form** | **√** |  |  |  |
| **Patient Data** | **√** |  |  |  |
| **Medical Checkup** | **√** | **√** | **√** | **√** |
| **Physical Examination** | **√** | **√** | **√** | **√** |
| **App Download (Test Group)** | **√** |  |  |  |
| **Training for App Use (Test Group)** | **√** | **√** | **√** | **√** |
| **MEMS^®^ Distribution** | **√** |  |  |  |
| **Training for MEMS^®^** | **√** | **√** | **√** | **√** |
| **Blood Test** | **√** |  |  | **√** |
| **Blood Chemistry Test** | **√** |  |  | **√** |
| **Estimated GFR (by MDRD)** | **√** |  |  | **√** |
| **Acute Rejection** | **√** | **√** | **√** | **√** |
| **Therapeutic Drug Monitoring** | **√** | **√** | **√** | **√** |
| **HAD-K, BFI-K-10** | **√** |  |  |  |
| **BAASIS with VAS** | **√** | **√** | **√** | **√** |
| **Electronic Monitoring** | **√** | **√** | **√** | **√** |

- The clinical test items include the following:

• Blood test: WBC with differential count, Hemoglobin, Hematocrit, RBC, Platelets
• Blood chemistry test: Renal function test (sodium, potassium, calcium, phosphorus, BUN, Creatinine), glucose, uric acid, AST, ALT, ALP, albumin, total bilirubin

• Serious adverse events (SAE):
 ▪ A fatal or life threatening event
 ▪ An event resulting in continuous or serious disability or incompetency
 ▪ An event requiring hospitalization or prolonged length of stay
 ▪ A medical emergency case (where medical or surgical treatment is required)
• Acute rejection refers solely to cases diagnosed by histological examination. Renal histological examination to confirm acute rejection may be carried out on cases showing two or more consecutive rises over 30 % of the baseline serum creatinine or where a medical professional admits the necessity of the examination.

• HAD scale

**Hospital Anxiety and Depression Scale**

Age: Gender: male / female

*Your emotional state may have substantial influence on your health or the disorder.

*Read each item and circle the reply that comes closest to how you have been feeling.


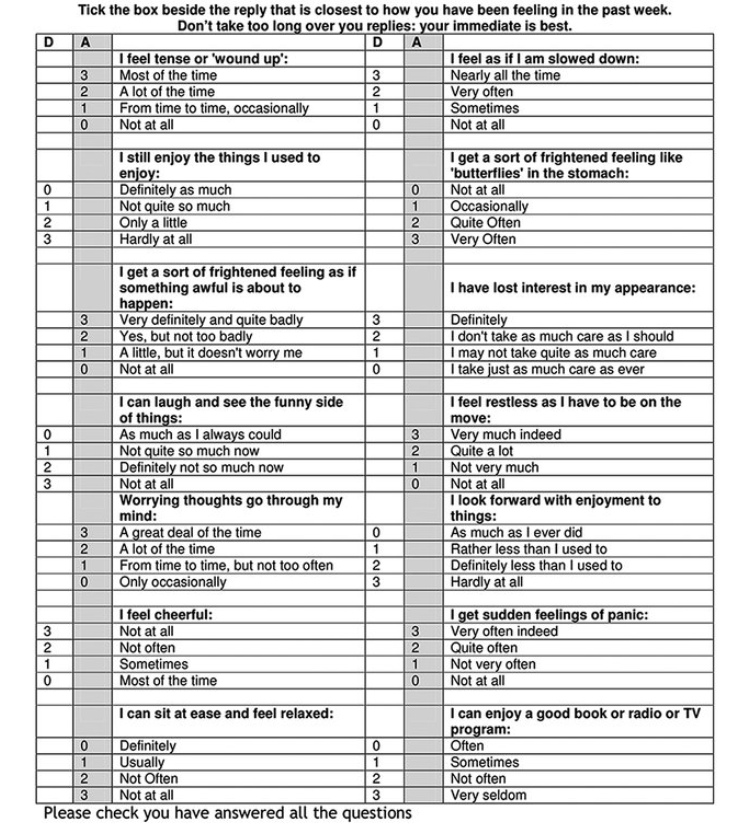


• BFI-K-10

**Big Five Inventory – Korean Version (BFI-K-10)**

Here are some questions that may relate to your personality. For each item

I am about to describe to you, reply with a score that best represents your personality.

| 1  Not at all | 2  Not so much | 3  Normally | 4  Frequently | 5  Definitely |
| --- | --- | --- | --- | --- |

“I consider myself to be__________.”

1. *__________ conventional/conservative

2. ___________ trustworthy

3. *__________ lazy

4. *__________ easygoing (capable of managing stress easily)

5. *__________ unartistic (having little interest in art)

6. ___________ outgoing and sociable

7. *__________ critical of others

8. ___________ thorough in the given tasks

9. ___________ irritable

10. __________ imaginative

• Basel Assessment of Adherence with Immunosuppressive medication scale (BAASIS) (Schafer-Keller P et al Am J Transplant 2008, Deschamps AE et al AIDS Patient Care STDS 2004, Kerr T et al AIDS Care 2005)

: Assessment of adherence over the past four weeks; 1-5 is defined as nonadherence

**<Basel Assessment of Adherence with Immunosuppressive medication scale (BAASIS)>**

| Question | 0  Never | 1 | 2 | 3 | 4 | 5  Always |
| --- | --- | --- | --- | --- | --- | --- |
| On one or several days in the past four weeks, I have forgotten to take the immunosuppressant. |  |  |  |  |  |  |
| On one or several days in the past four weeks, I have taken the immunosuppressant two hours before or after the prescribed time of medication. |  |  |  |  |  |  |
| On two or more consecutive days in the past four weeks, I have forgotten to take the immunosuppressant. |  |  |  |  |  |  |
| On one or several days in the past four weeks, I have taken the immunosuppressant in the dose lower than the prescribed dose. |  |  |  |  |  |  |

• VAS (Visual analogue scale)

전혀 복용 안함: Never took any medication

완벽히 복용함: Took all medications perfectly


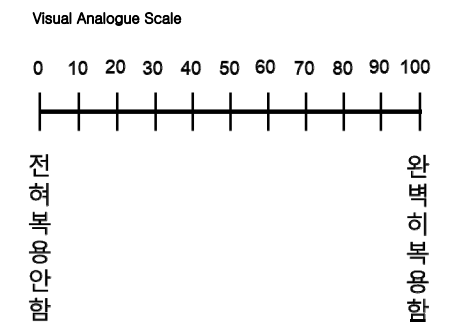


• MEMS ® parameters: Automatically extracted using MEMS®

1) Taking adherence: percentage of cap removals compared with the number prescribed for the monitoring period
2) Dosing adherence: percentage of days on which the patient took the prescribed number of doses
3) Timing adherence: percentage of correct dosing intervals (**±** 1 hour of prescribed intake timing)
4) Number of drug holidays: no cap removals over a period greater than 24 h

1. **Adherence assessment**

The assessment of medication adherence will be based on MEMS® and BAASIS. On the day of screening visit, the patient will be trained in the use of MEMS®, and for the test group, the mobile internet application will be installed on the patient’s phone and the patient trained in its use. On Day 28 (Study visit 1), current use of MEMS® and the app will be checked and retraining provided. On Day 90 (Study visit 2), current use of MEMS® and the app will be checked once again. On this day, the adherence rate recorded by MEMS® will not be disclosed to the patient, while (s)he will be informed of the cases of inappropriate use and retraining provided.

1. **Methods and variables of efficacy evaluation and reporting**

To verify whether the use of the mobile internet application has led to improved adherence to immunosuppressants, the following will be assessed:

- Primary endpoint: Evaluation of the differences in 6 months nondherence rate (assessed by MEMS®) between groups after the onset of study.

• Nonadherence: a taking adherence of <98% or >102% and/or at least one drug holiday

- Secondary endpoints: Determination of the factors that influence the adherence rate based on demographic, socioeconomic, medical, and surgical factors as well as those identified by HAD-K and BFI-K-10, for which the Collateral monitoring (BAASIS with 120 mm VAS, tacrolimus or cyclosporine trough level, and the adherence rate on the app) and MEMS® are used to evaluate the correlations among the assessed adherence rates and the variations in the incidence of acute rejection and eGFRMDRD between the groups.

1. **Statistical analysis: principles and methods**

MRCC was consulted for the methods of randomization and statistical analysis.

1) Subject group
The data analysis of the differences in the rate of incidence of acute rejection and the variations in eGFRMDRD, among primary and secondary efficacy evaluation items, requires randomization and non-violation of the protocol, and targets the patients per protocol (PP) who have completed all visits during the six-month period. Also, the secondary efficacy evaluation targets all randomized patients who used MEMS®.

2) Statistical analysis
The differences in adherence rate between groups will be compared using a chi-square test. Subjects will be grouped by adherence rate (>95-100%, 80-95%, 50-80%, and 0-50%), to evaluate the factors influencing adherence including demographic, socioeconomic, medical, and surgical factors, based on a multivariable ordinal logistic regression model. SPSS version 17.0 will be used.

1. **Data and Safety Monitoring Plan**
2. **Monitoring**
   The participants of the study should review the clinical study protocol and the case report at the initiation meeting prior to the onset of study. During the study period, the sub-investigator will check the completeness of patient records, the accuracy of the case reports, and the clinical study protocol and enrollment status, every three months until the termination of study in order to make sure that the study is in compliance with the protocol. The investigator will maintain the documents containing the demographic, medical, and lab data, and a record of other test results and evaluation results, per each subject. All data included in the case report should be traceable based on the documents in each subject’s file. The investigator will store the original copy of the informed consent form signed by the subject, while a single copy of the signed form will be given to the subject. The monitoring criteria requires the standard verification of all data that may be used as a variable in efficacy evaluation, as well as keeping the consent forms, the compliance to inclusion/exclusion criteria, and the record of SAEs. No data in the evidential documents regarding subject identity may be disclosed.
3. **Data storage**
   The investigator should maintain an appropriate and accurate record of the contents of the study to allow future access to any of the data related to the study. All data and documents related to the study should be stored in a place with limited access, open only to IRB personnel or relevant staff. The documents related to the study should be stored in files for a minimum of ten years after the completion or termination of study. The documents may be discarded once the period of ten years has expired.
4. **Privacy protection for subjects**

To prevent any leakage of personal information due to the loss of the smartphone, etc., the original data may be deleted via the internet.

1. **Study protocol schedule**

1) Date of IRB approval – 2 years post-IRB approval
2) For 3 months post-IRB approval: Development of the mobile internet application
3) Month 3 to 4 post-IRB approval: Test trial of the application
4) Month 4 to 24 post-IRB approval: Patient enrollment
5) Month 4 to 30 post-IRB approval: End of patient follow-up
6) Month 30 to 36 post-IRB approval: Data interpretation and research presentation

1. **Other ethical and scientific considerations**

The plans and execution of the present study shall comply with the latest Declaration of Helsinki revised in the 59^th^ World Medical Association (WMA) Seoul Conference in 2008, whereby all data collected for the study shall be safely managed so as to prevent leakage and no personally identifying data shall be collected. Furthermore, the study shall strictly comply with the IRB ethics. The data obtained through the application shall also be safely managed to prevent leakage and no personally identifying data shall be collected.

1. **References**

1) Cramer JA, Roy A, Burrell A, et al. Medication compliance and persistence: terminology and definitions. Val Health 2008;11: 44-47.
2) DiMatteo MR. Variations in Patients’ adherence to medical recommendations. A quantitative review of 50 years of research. Medical Care 2004;42:200-209.
3) Fine RN, Becker Y, Geest SD, et al. Nonadherence consensus conference summary report. Am J Transplant 2009;9:35-41.
4) Weng FL, Israni AK, Joffe MM, et al. Race and electronically measured adherence to immunosuppressive medications after deceased donor renal transplantation. J Am Soc Nephrol 2005;16:1839-1848.
5) Belser LD, et al. Interventions to improve medication adherence after transplantation: a systematic review. Transplant international 2009;22:780.
6) Oh SM, et al. A study on the standardization of the hospital anxiety and depression scale for Koreans. J Korean Neuropsychiatr Assoc 1999;38:289.
7) Kim SY, et al. Standardization and validation of big five inventory-korean version (BFI-K) in elders. Korean Journal of Biological Psychiatry 2010;17:15.
8) Schafer-Keller P, Steiger J, Bock A et al. Diagnostic accuracy of measurement methods to assess non-adherence to immunosuppressive drugs in kidney transplant recipients. Am J Transplant 2008; 8: 616.
9) Deschamps AE, Grave VD, Van Wijngaerden Et, et al. Prevalence and correlates of nonadherence to antiretroviral therapy in a population of HIV patients using Medication Event Monitoring System. AIDS Patient Care STDS 2004;18:644.
10) Kerr T, Marshall A, Walsh J, et al. Determinants of HAART discontinuation among injection drug users. AIDS Care 2005;17:539.

**Informed Consent Form**

**Improving Adherence to Immunosuppressive Therapy by**

**Mobile Internet Application in Solid Organ Transplant Patients**

1. I have been given a verbal explanation regarding the clinical study and read the attached information regarding participation in the clinical study, for which I have had a discussion with the investigator in charge.

2. I have been informed of the risks and benefits, and any questions I have asked, have been answered to my satisfaction.

3. I voluntarily consent to participate in this study.

4. I am aware that I may refuse to participate in this study or withdraw from it at any time and that such a decision shall not affect future treatment nor inflict any harm.

5. I agree that by signing this consent form and the attached information sheet, I consent to the collection of my personal information and its use for medical purposes by the investigator, within the protection of current laws and regulations.

6. I am aware that I shall be given a copy of the consent form and the attached information.

Patient name signature date

(For patients below the age of 15 - 18)

Legal guardian name signature date

(Relation: )

Co-investigator name signature date
(Sub-investigator or Principal Investigator)

**Description of the Clinical Study**

**(for participant)**

**Title:**

Improving Adherence to Immunosuppressive Therapy using a Mobile Internet Application in Solid Organ Transplant Patients

**Background and purpose:**

Adherence is an indicator of how accurately a patient takes the prescribed dose of immunosuppressant medication at the correct times after organ transplantation. It has a profound influence on the prognosis of the transplanted organ. In cases where irregular doses of immunosuppressants result in low suppression of immunity, acute or chronic rejection, or antibody-mediated rejection due to released antibodies may result, this induces chronic organ damage and eventually leads to the loss of the transplanted organ.

Nonetheless, previous studies on transplant patients reported 40 – 70 % adherence, which provided the basis for planning the present study, where a novel mobile internet application has been developed to improve adherence to either Tacrolimus or Cyclosporine, the principal immunosuppressants for transplant patients.

This application has been designed to notify you of the time to take Tacrolimus or Cyclosporine by an alarm and to allow you to check when you took your medication and share this data with your physician.

**Monitoring methods and monitored items:**

Upon your consent for participation in this study, a simple questionnaire on the current status of medication (BAASIS), personality, and depression will be given to you to complete as a participant. A cap and a container of an electronic pill box (MEMS®) will be provided, with guidelines and precautions on its use. In the future, Tacrolimus or Cyclosporine is to be stored in this pill box and opening the cap will result in a record of the time you took the medication, this data will be downloaded at the hospital upon your subsequent outpatient visit.

The participants in the present study are randomly assigned to a group (69 subjects) requested to use the mobile internet application and a control group (69 subjects). If you have been allocated to the application user group, first, the app will be installed on your smartphone, and the investigators will do their best to explain to you and help you with the installation and use of the app; then, during the six-month study period, you will be requested to use the app as you take either Tacrolimus or Cyclosporine stored in the MEMS®. If you have been allocated to the control group, you will be requested to take either Tacrolimus or Cyclosporine stored in MEMS® without the use of the application.

As you participate in this study, you will be requested to visit the hospital on Days 28, 90, and 180, to estimate the drug concentration and to receive a renal function test and complete the questionnaire for medication (BAASIS).

**Predicted benefits:**

By participating in this study, you will be given an opportunity to benefit from the use of a globally recognized and high-cost MEMS® device for checking your adherence to immunosuppressants. In the case of low adherence, consultations on medication may improve the adherence to help maintain reliable function of the transplanted kidney for prolonged time.

**Financial aspects of participation:**

By participating in the present study, you will be subject to no additional direct cost. Nonetheless, there may be a slight increase in your phone bill due to the installation and use of the mobile internet application, which will be compensated for by a fixed participation fee (50,000 won) on the completion of the study.

**Possible inconvenience:**

As you participate in this study, you will be requested to store Tacrolimus or Cyclosporine in a MEMS® and follow the prescribed medication. In addition, the cap of the pill box should only be opened when taking medication and on no other occasions.

The use of the mobile internet application for checking the medication may cause a slight inconvenience.

**Voluntary participation:**

Participation in this study should be determined voluntarily, and there will be no disadvantage incurred by choosing not to participate in the study. You may decide not to participate in the study or withdraw from the study at any time. Furthermore, such decision will not affect your future treatments. At any time you may consult a physician or nurse regarding any inquiries you may have on the present study and your rights as a participant. In addition, if new information is collected that may influence your decision regarding continuing participation in the study, you or your guardian will be informed immediately.

**Drop-out cases and rationale:**

In the following cases, you may be withdrawn from the study without your consent:

- Graft loss

- Death

- Significant violation of the protocol

- Early termination of the study

**Confidentiality:**

All data regarding the subjects of this study are strictly confidential, and therefore, all data are labeled solely with a number. The code number is disclosed to no one but the participant and the principal investigator. The data will be used only for the purpose of writing and submitting the academic paper; no data that may identify patients will be included in the paper, and all data will be presented only after it has been processed and analyzed, then integrated. However, in an unavoidable circumstance that requires the disclosure of the code, an appropriate legal procedure will be followed. The data obtained during this study may continue to be used to improve upon this study in the future; hence, the data will not be discarded during the period agreed upon by the consent holder, and all data will be stored under the supervision of the principal investigator who takes the full responsibility.

**Based on a thorough understanding and consideration of the above facts, please determine your participation in the present study. Upon your consent for participation, you will be provided with a leaflet containing the description of the clinical study and a copy of the informed consent form. Should you wish to obtain additional information regarding the rights and benefits of the study or the participants, or when damage is caused by the study, please contact the number below:**

**Seoul National University Hospital IRB +82 (0)2 2072 0694**
