# Supplementary material for: Mobile medication manager application to improve adherence with immunosuppressive therapy in renal transplant recipients: A randomized controlled trial
Source: PLoS One. 2019 Nov 5;14(11):e0224595. doi: 10.1371/journal.pone.0224595 (PMC6830819; doi:10.1371/journal.pone.0224595)
Supplement: S1 Fig — (PPTX) [file pone.0224595.s002.pptx]

## Slide 1
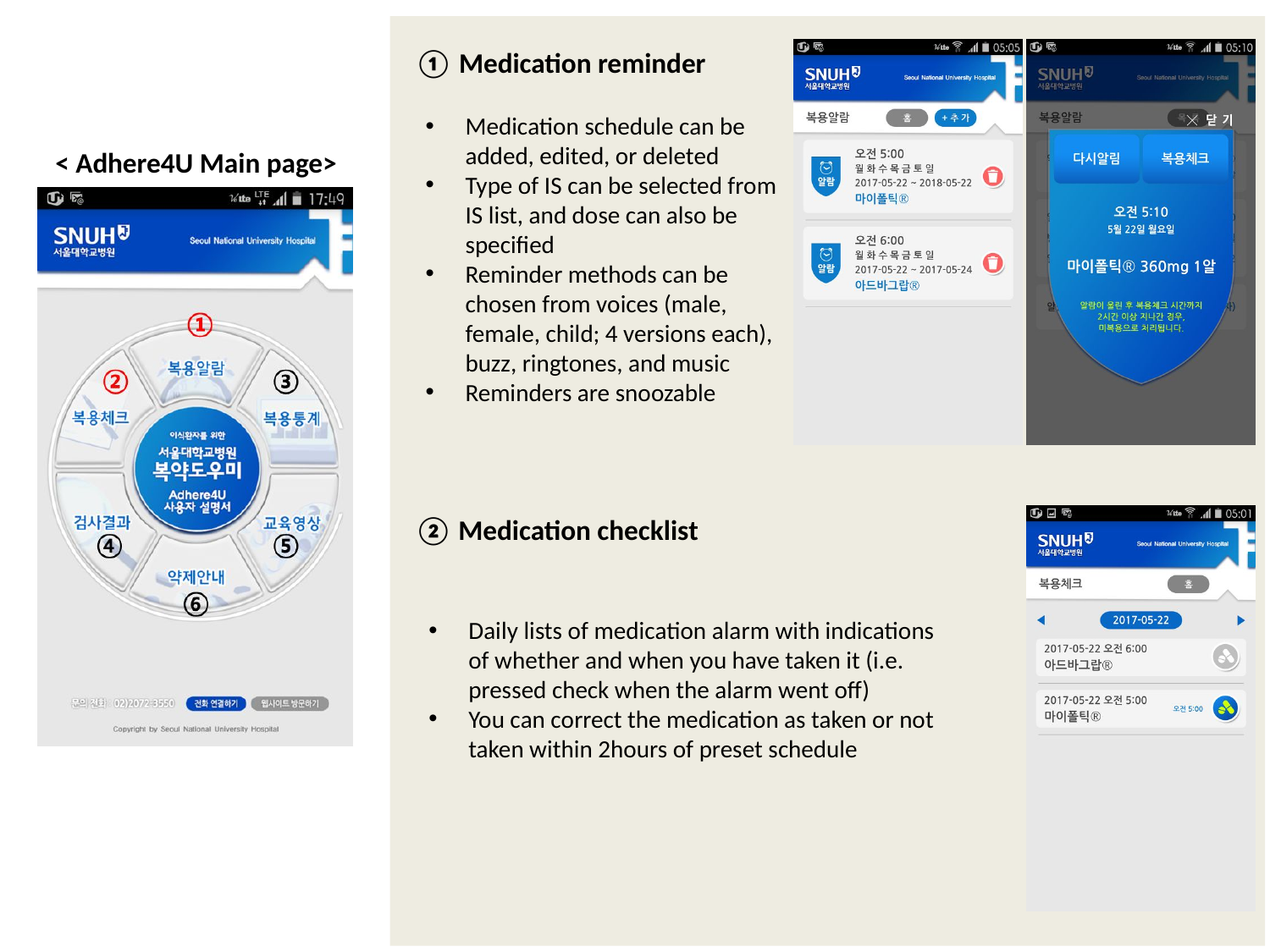

① Medication reminder
Medication schedule can be added, edited, or deleted
Type of IS can be selected from IS list, and dose can also be specified
Reminder methods can be chosen from voices (male, female, child; 4 versions each), buzz, ringtones, and music
Reminders are snoozable
< Adhere4U Main page>
② Medication checklist
Daily lists of medication alarm with indications of whether and when you have taken it (i.e. pressed check when the alarm went off)
You can correct the medication as taken or not taken within 2hours of preset schedule

## Slide 2
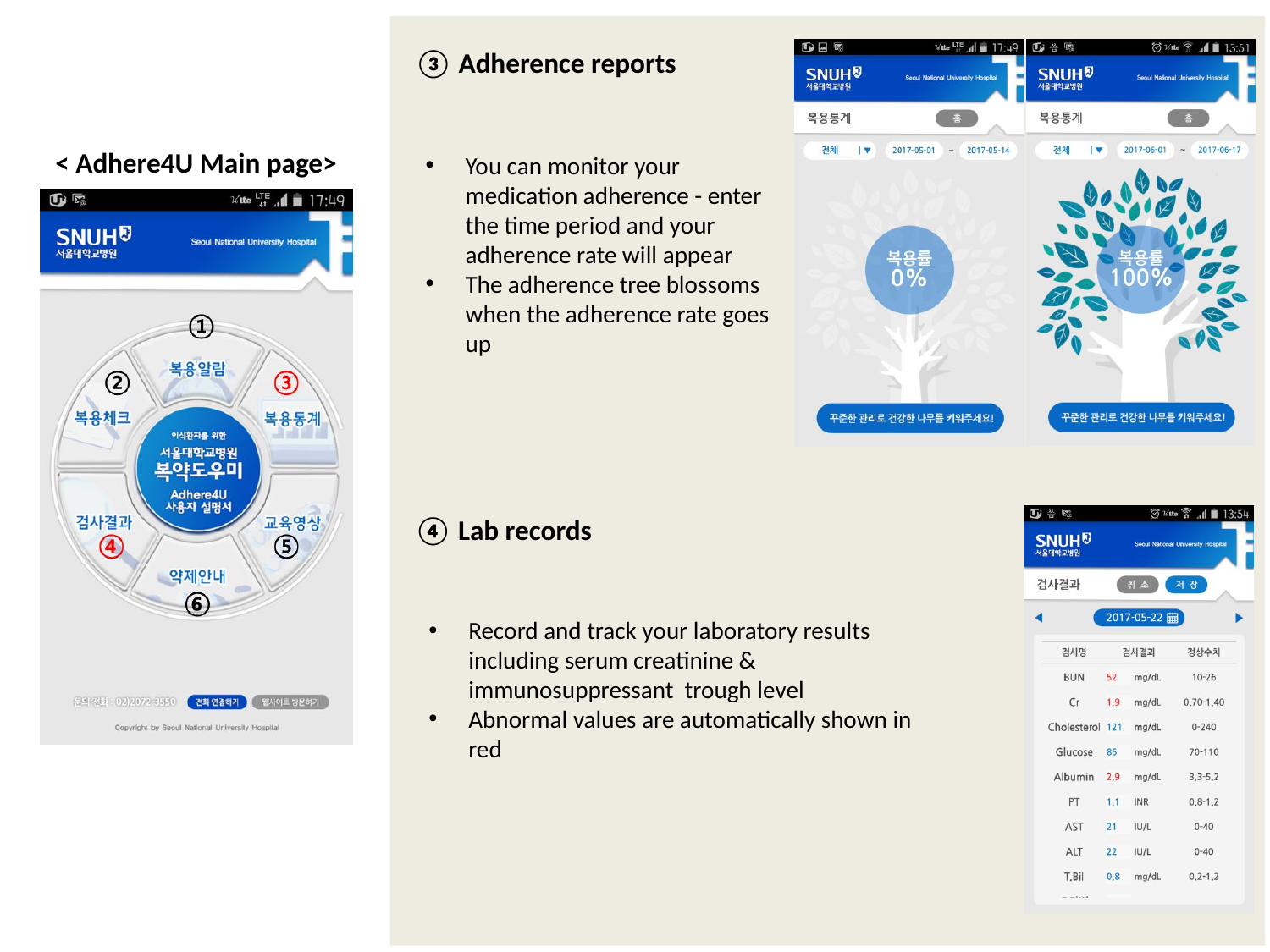

③ Adherence reports
< Adhere4U Main page>
You can monitor your medication adherence - enter the time period and your adherence rate will appear
The adherence tree blossoms when the adherence rate goes up
④ Lab records
Record and track your laboratory results including serum creatinine & immunosuppressant trough level
Abnormal values are automatically shown in red
⑥

## Slide 3
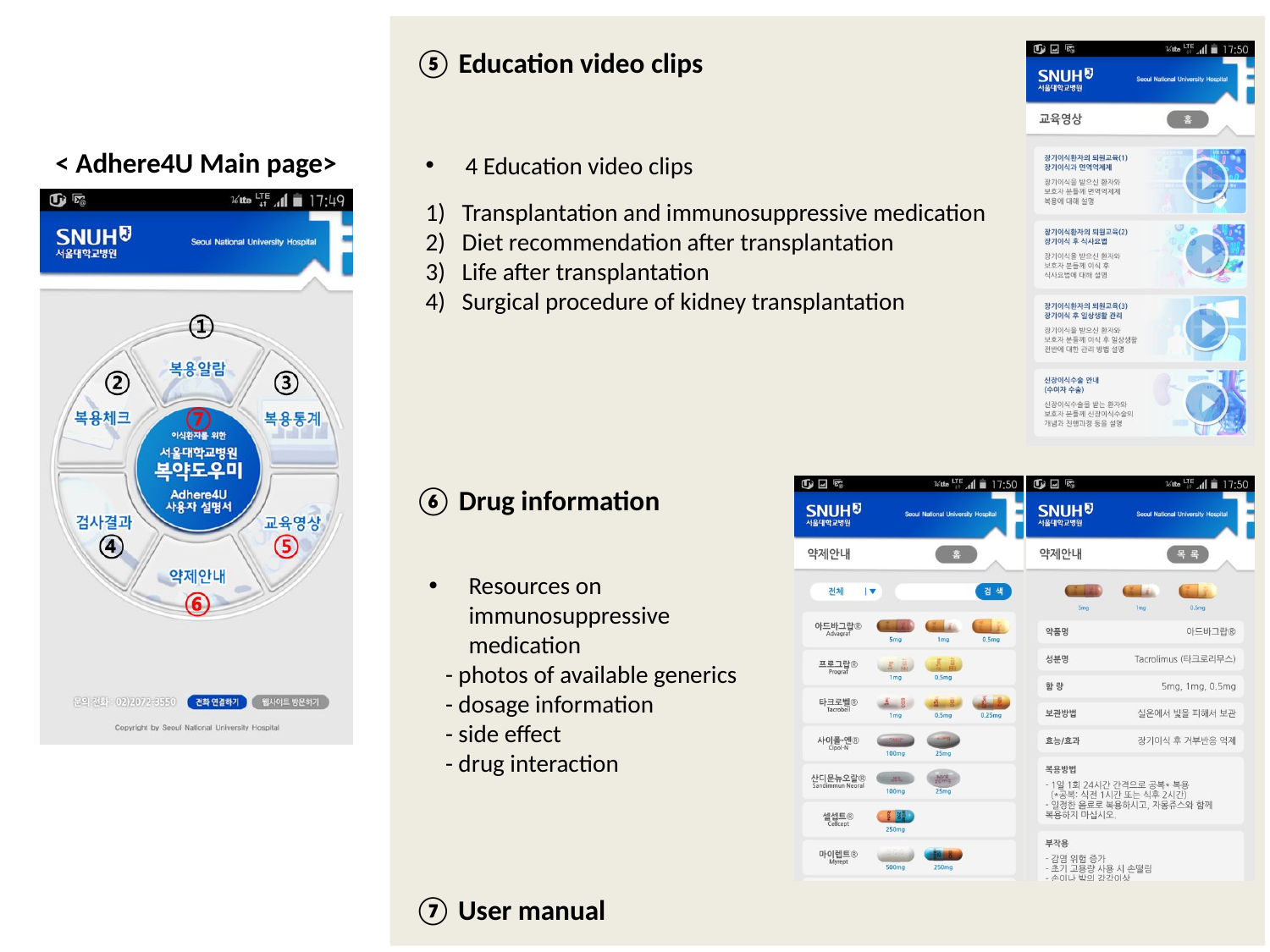

⑤ Education video clips
< Adhere4U Main page>
4 Education video clips
1) Transplantation and immunosuppressive medication
2) Diet recommendation after transplantation
3) Life after transplantation
4) Surgical procedure of kidney transplantation
⑥ Drug information
Resources on immunosuppressive medication
 - photos of available generics
 - dosage information
 - side effect
 - drug interaction
⑦ User manual
