# Supplementary material for: Mobile medication manager application to improve adherence with immunosuppressive therapy in renal transplant recipients: A randomized controlled trial
Source: PLoS One. 2019 Nov 5;14(11):e0224595. doi: 10.1371/journal.pone.0224595 (PMC6830819; doi:10.1371/journal.pone.0224595)
Supplement: S2 Fig — Comparison of daily percentage of patients with (A) correct dosing and (B) correct timing. (PPTX) [file pone.0224595.s003.pptx]

## Slide 1
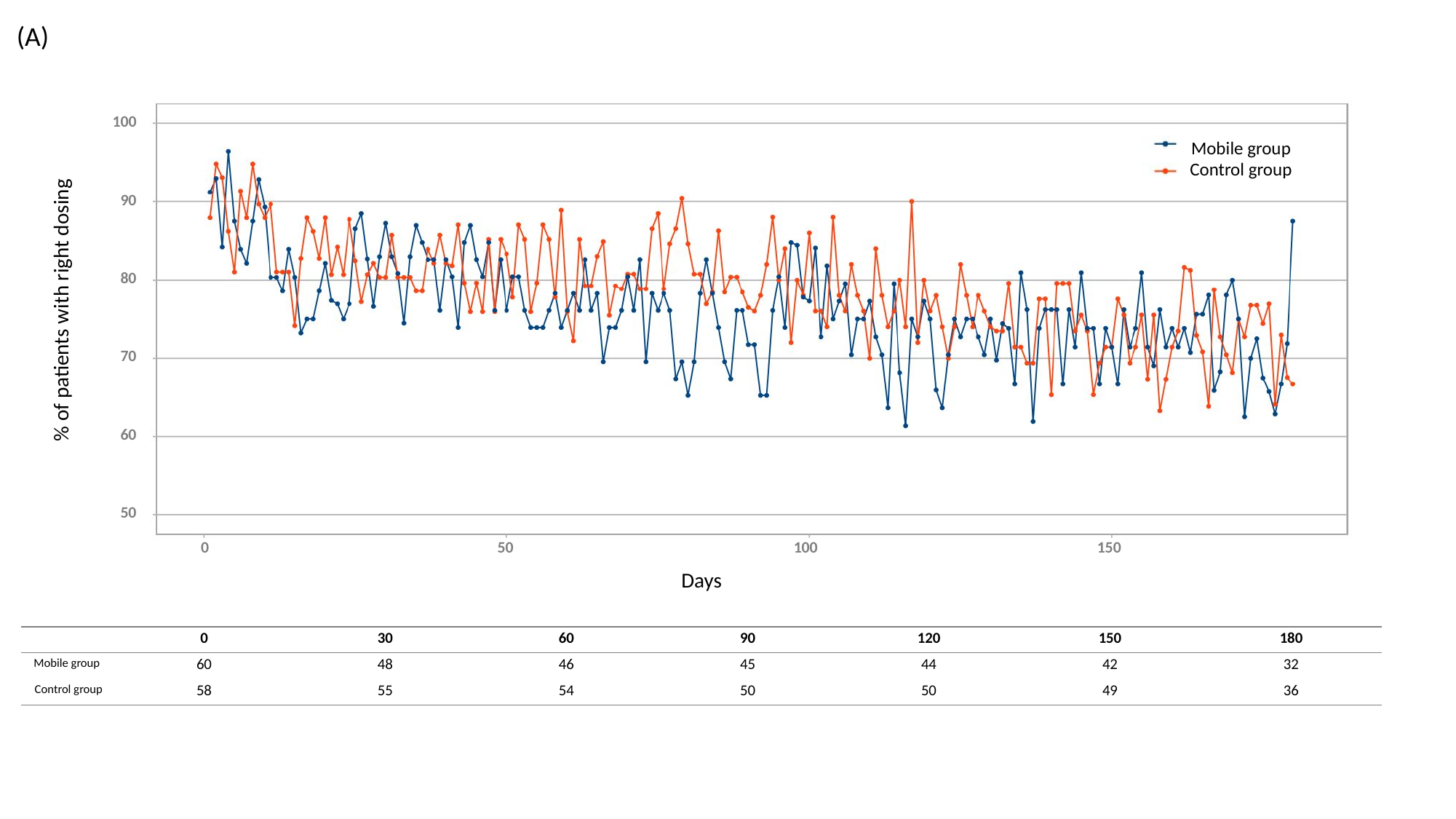

(A)
| 100 |
| --- |
| 90 |
| 80 |
| 70 |
| 60 |
| 50 |
Mobile group
Control group
% of patients with right dosing
| 0 | 50 | 100 | 150 |
| --- | --- | --- | --- |
Days
| | 0 | 30 | 60 | 90 | 120 | 150 | 180 |
| --- | --- | --- | --- | --- | --- | --- | --- |
| Mobile group | 60 | 48 | 46 | 45 | 44 | 42 | 32 |
| Control group | 58 | 55 | 54 | 50 | 50 | 49 | 36 |

## Slide 2
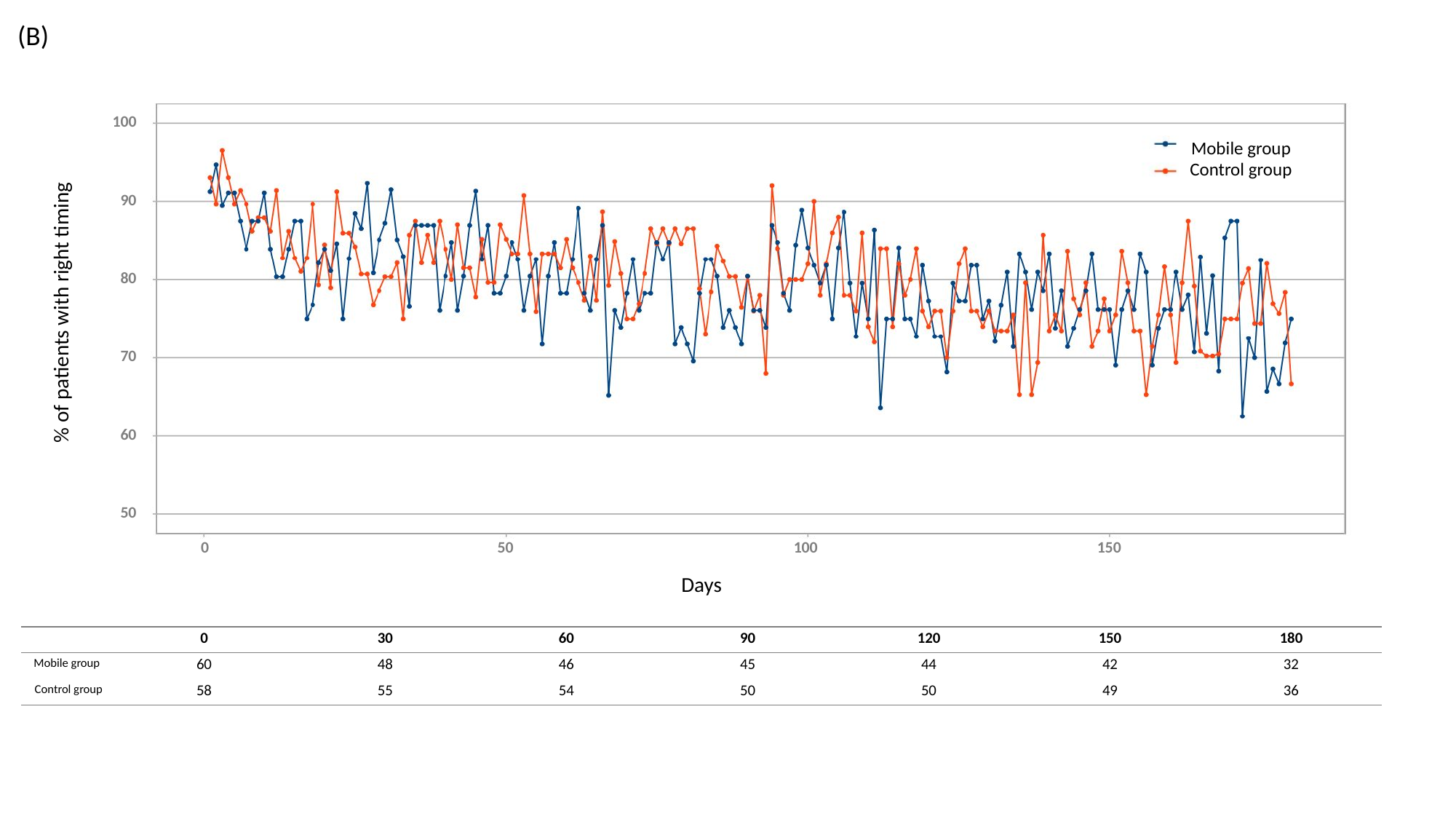

(B)
| 100 |
| --- |
| 90 |
| 80 |
| 70 |
| 60 |
| 50 |
Mobile group
Control group
% of patients with right timing
| 0 | 50 | 100 | 150 |
| --- | --- | --- | --- |
Days
| | 0 | 30 | 60 | 90 | 120 | 150 | 180 |
| --- | --- | --- | --- | --- | --- | --- | --- |
| Mobile group | 60 | 48 | 46 | 45 | 44 | 42 | 32 |
| Control group | 58 | 55 | 54 | 50 | 50 | 49 | 36 |
