# Supplementary material for: Mobile medication manager application to improve adherence with immunosuppressive therapy in renal transplant recipients: A randomized controlled trial
Source: PLoS One. 2019 Nov 5;14(11):e0224595. doi: 10.1371/journal.pone.0224595 (PMC6830819; doi:10.1371/journal.pone.0224595)
Supplement: S1 Table — (DOCX) [file pone.0224595.s004.docx]

**S1 Table. Baseline characteristics of the patients included and excluded in the analysis**

|  | **Total (*n* = 136)** | **Included**  **(*n* = 118)** | **Excluded**  **(*n* = 18)** | **P-value** |
| --- | --- | --- | --- | --- |
| ***Sociodemographics*** |  |  |  |  |
| Age (years), median (IQR) | 43.0 (34.0–53.0) | 43.0 (35.0–53.0) | 44.5 (29.0–53.0) | 0.74 |
| BMI (kg/m^2^), mean±SD | 22.2 ± 3.1 | 22.1 ± 3.1 | 22.5 ± 3.1 | 0.58 |
| Male sex, *n* (%) | 88 (64.7) | 76 (64.4) | 12 (66.7) | 1.00 |
| Education level, *n* (%) |  |  |  | 0.72 |
| Less than middle school | 7 (5.1) | 6 (5.1) | 1 (5.6) |  |
| Middle school | 19 (14.0) | 15 (12.7) | 4 (22.2) |  |
| Highschool | 48 (35.3) | 43 (36.4) | 5 (27.8) |  |
| University | 62 (45.6) | 54 (45.8) | 8 (44.4) |  |
| Occupation, *n* (%) |  |  |  | 0.77 |
| Full time | 67 (49.3) | 57 (48.3) | 10 (55.6) |  |
| Part time | 9 (6.6) | 9 (7.6) | 0 |  |
| Student | 13 (9.6) | 11 (9.3) | 2 (11.1) |  |
| Housewife | 27 (19.9) | 23 (19.4) | 4 (22.2) |  |
| Unemployed | 21 (15.4) | 18 (15.3) | 3 (16.7) |  |
| Smoking, *n* (%) |  |  |  | 0.67 |
| Current smoker | 4 (2.9) | 4 (3.4) | 0 |  |
| Previous smoker | 1 (0.7) | 1 (0.8) | 0 |  |
| Non smoker | 131 (96.3) | 113 (95.8) | 18 (100.0) |  |
| ***Clinical characteristics*** |  |  |  |  |
| Causes of ESRD, *n* (%) |  |  |  | 0.65 |
| IgA nephropathy | 30 (22.1) | 24 (20.3) | 6 (33.3) |  |
| Glomerulonephritis | 16 (11.8) | 13 (11.0) | 3 (16.7) |  |
| ADPKD | 15 (11.0) | 14 (11.9) | 1 (5.6) |  |
| Hypertension | 8 (5.9) | 8 (6.8) | 0 |  |
| Diabetes | 8 (5.9) | 6 (5.1) | 2 (11.1) |  |
| FSGS | 8 (5.9) | 6 (5.1) | 2 (11.1) |  |
| Vesicoureteral reflux | 6 (4.4) | 5 (4.2) | 1 (5.6) |  |
| SLE | 5 (3.7) | 5 (4.2) | 0 |  |
| HSN | 4 (2.9) | 4 (3.4) | 0 |  |
| unknown | 29 (21.3) | 27 (22.9) | 2 (11.1) |  |
| others | 7 (5.1) | 6 (5.1) | 1 (5.6) |  |
| Dialysis before transplantation, *n* (%) | 114 (83.8) | 98 (83.1) | 16 (88.9) | 0.78 |
| Dialysis duration (months), median (IQR) | 28.5 (3.0–67.3) | 26.1 (2.7–63.4) | 37.7 (19.1–81.8) | 0.13 |
| Time since transplantation (months), median (IQR) | 24.6 (13.6–53.2) | 25.2 (13.7–52.2) | 17.3 (12.7–59.1) | 0.32 |
| Donor type, *n* (%) |  |  |  | 0.12 |
| Living donor |  |  |  |  |
| - 1^st^ degree related | 28 (20.6) | 24 (20.3) | 4 (22.2) |  |
| - other related | 30 (22.1) | 28 (23.7) | 2 (11.1) |  |
| - spouse | 20 (14.7) | 20 (16.9) | 0 |  |
| - other nonrelated | 1 (0.7) | 1 (0.8) | 0 |  |
| Deceased donor | 57 (41.9) | 45 (38.1) | 12 (66.7) |  |
| Number of transplantation, *n* (%) |  |  |  | 0.64 |
| First | 128 (94.1) | 112 (94.9) | 16 (88.9) |  |
| Second | 8 (5.9) | 6 (5.1) | 2 (11.1) |  |
| Number of IS, *n* (%) |  |  |  | 0.71 |
| 2 | 23 (16.9) | 21 (17.8) | 2 (11.1) |  |
| 3 | 113 (83.1) | 97 (82.2) | 16 (88.9) |  |
| Type of calcineurin inhibitor, *n* (%) |  |  |  | 1.00 |
| Cyclosporine A | 8 (5.9) | 7 (5.9) | 1 (5.6) |  |
| Tacrolimus | 128 (94.1) | 111 (94.1) | 17 (94.4) |  |
| Number of medication other than IS, median (IQR) | 3.0 (2.0–5.0) | 3.0 (2.0–5.0) | 4.5 (3.0–6.0) | 0.03 |
| Previous acute rejection, *n* (%) |  |  |  | 0.59 |
| None | 94 (69.6) | 84 (71.8) | 10 (55.6) |  |
| 1 | 31 (23.0) | 25 (21.4) | 6 (33.3) |  |
| 2 | 8 (5.9) | 6 (5.1) | 2 (11.1) |  |
| ≥ 3 | 2 (1.4) | 2 (1.8) | 0 |  |
| Serious infection after transplantation, *n* (%) | 25 (18.4) | 23 (19.5) | 2 (11.1) | 0.60 |
| Systolic blood pressure (mmHg), mean±SD | 122.6 ± 10.2 | 122.9 ± 10.7 | 120.9 ± 6.8 | 0.32 |
| Serum creatinine (mg/dl), mean±SD | 1.3 ± 0.3 | 1.2 ± 0.3 | 1.4 ± 0.3 | 0.12 |
| MDRD GFR (ml/min/1.73 m^2^), median (IQR) | 63.0 (53.4–74.0) | 63.2 (53.6–75.5) | 57.3 (48.7–68.0) | 0.11 |
| 6 mo. intraindividual variability of CNI, median (IQR) | 13.0 (8.6–18.8)^a^ | 12.8 (8.1-18.5) | 14.7 (9.3-23.3) | 0.66 |
| HADS anxiety score, median (IQR) | 5 (3–7) | 5 (3–7) | 5 (2–7) | 0.77 |
| HADS depression score, median (IQR) | 6 (4–8) | 6 (4–8) | 5.5 (4–7) | 0.94 |
| BFI-10 neuroticism score, median (IQR) | 3.0 (2.0–3.5) | 2.8 (2.0–3.5) | 3.0 (2.5–3.0) | 0.82 |
| BFI-10 openness score, median (IQR) | 3.5 (3.0–4.0) | 3.5 (3.0–4.0) | 3.5 (2.5–4.0) | 0.73 |
| BFI-10 extraversion score, median (IQR) | 3.0 (2.5–3.3) | 3.0 (2.8–3.3) | 2.8 (2.2–3.0) | 0.07 |
| BFI-10 agreeableness score, median (IQR) | 3.5 (3.0–4.0) | 3.5 (3.0–4.0) | 3.5 (3.5–4.0) | 0.12 |
| BFI-10 conscientiousness score, median (IQR) | 3.5 (3.0–4.0) | 3.5 (3.0–4.0) | 4.0 (3.0–4.5) | 0.35 |

SD, standard deviation; BMI, body mass index; ESRD, end stage renal disease; IgA, immunoglobulin A; ADPKD, autosomal dominant polycystic kidney disease; FSGS, focal segmental glomerulosclerosis; SLE, systemic lupus erythematosus; HSN, Henoch Schönlein nephritis; IS, immunosuppressant; IQR, interquartile range; MDRD GFR, glomerular filtration rate by Modification in Diet in Renal Disease study equation; CNI, calcineurin inhibitor; HADS, Hospital Anxiety and Depression Scale; BFI-10, 10-item Big Five Inventory; BAASIS, Basel Assessment of Adherence to Immunosuppressive Medication Scale; VAS, Visual Analog Scale.

^a^ values missing in 4 patients
