# Supplementary material for: Mobile medication manager application to improve adherence with immunosuppressive therapy in renal transplant recipients: A randomized controlled trial
Source: PLoS One. 2019 Nov 5;14(11):e0224595. doi: 10.1371/journal.pone.0224595 (PMC6830819; doi:10.1371/journal.pone.0224595)
Supplement: S2 Table — (DOCX) [file pone.0224595.s005.docx]

**S2 Table. Characteristics of the two study group population included in the analysis for primary end point (n=118)**

|  | **Total (*n* = 118)** | **Control**  **(*n* = 58)** | **Mobile**  **(*n* = 60)** | **P-value** |
| --- | --- | --- | --- | --- |
| ***Sociodemographics*** |  |  |  |  |
| Age (years), mean±SD | 43.2 ± 12.6 | 41.3 ± 13.5 | 45.1 ± 11.5 | 0.10 |
| BMI (kg/m^2^), mean±SD | 22.2 ± 3.1 | 22.1 ± 3.1 | 22.5 ± 3.1 | 0.58 |
| Male sex, *n* (%) | 76 (64.4) | 40 (69.0) | 36 (60.0) | 0.41 |
| Education level, *n* (%) |  |  |  | 0.49 |
| Less than middle school | 6 (5.1) | 4 (6.9) | 2 (3.3) |  |
| Middle school | 15 (12.7) | 5 (8.6) | 10 (16.7) |  |
| Highschool | 43 (36.4) | 21 (36.2) | 22 (36.7) |  |
| University | 54 (45.8) | 28 (48.3) | 26 (43.3) |  |
| Occupation, *n* (%) |  |  |  | 0.53 |
| Full time | 57 (48.3) | 26 (44.8) | 31 (51.7) |  |
| Part time | 9 (7.6) | 5 (8.6) | 4 (6.7) |  |
| Student | 11 (9.3) | 8 (13.8) | 3 (5.0) |  |
| Housewife | 23 (19.5) | 10 (17.2) | 13 (21.7) |  |
| Unemployed | 18 (15.3) | 9 (15.0) | 9 (15.3) |  |
| Smoking, *n* (%) |  |  |  | 0.36 |
| Current smoker | 4 (3.4) | 3 (5.2) | 1 (1.7) |  |
| Previous smoker | 1 (0.8) | 0 | 1 (1.7) |  |
| Non smoker | 113 (95.8) | 55 (94.8) | 58 (96.7) |  |
| ***Clinical characteristics*** |  |  |  |  |
| Causes of ESRD, *n* (%) |  |  |  | 0.65 |
| IgA nephropathy | 24 (20.3) | 15 (25.9) | 9 (15.0) |  |
| Glomerulonephritis | 13 (11.0) | 6 (10.3) | 7 (11.7) |  |
| ADPKD | 14 (11.9) | 6 (10.3) | 8 (13.3) |  |
| Hypertension | 8 (6.8) | 3 (5.2) | 5 (8.3) |  |
| Diabetes | 6 (5.1) | 3 (5.2) | 3 (5.0) |  |
| FSGS | 6 (5.1) | 3 (5.2) | 3 (5.0) |  |
| Vesicoureteral reflux | 5 (4.2) | 3 (5.2) | 2 (3.3) |  |
| SLE | 5 (4.2) | 4 (6.9) | 1 (1.7) |  |
| HSN | 4 (3.4) | 2 (3.4) | 2 (3.3) |  |
| unknown | 27 (22.9) | 9 (15.5) | 18 (30.0) |  |
| others | 6 (5.1) | 4 (6.9) | 2 (3.3) |  |
| Dialysis before transplantation, *n* (%) | 98 (83.1) | 51 (87.9) | 47 (78.3) | 0.25 |
| Dialysis duration (months), median (IQR) | 26.1 (2.7–63.4) | 24.5 (2.7–56.8) | 29.0 (2.2–69.3) | 0.79 |
| Time since transplantation (months), median (IQR) | 25.2 (13.7–52.2) | 23.8 (13.3–54.1) | 27.2 (14.0–47.7) | 0.50 |
| Donor type, *n* (%) |  |  |  | 0.83 |
| Living donor |  |  |  |  |
| - 1^st^ degree related | 24 (20.3) | 13 (22.4) | 11 (18.3) |  |
| - other related | 28 (23.7) | 13 (22.4) | 15 (25.0) |  |
| - spouse | 20 (16.9) | 9 (15.5) | 11 (18.3) |  |
| - other nonrelated | 1 (0.8) | 1 (1.7) | 0 |  |
| Deceased donor | 45 (38.1) | 22 (37.9) | 23 (38.3) |  |
| Number of transplantation, *n* (%) |  |  |  | 0.64 |
| First | 112 (94.9) | 54 (93.1) | 58 (96.7) |  |
| Second | 6 (5.1) | 4 (6.9) | 2 (3.3) |  |
| Number of IS, *n* (%) |  |  |  | 0.57 |
| 2 | 21 (17.8) | 12 (20.7) | 9 (15.0) |  |
| 3 | 97 (82.2) | 46 (79.3) | 51 (85.0) |  |
| Type of calcineurin inhibitor, *n* (%) |  |  |  | 0.13 |
| Cyclosporine A | 7 (5.9) | 1 (1.7) | 6 (10.0) |  |
| Tacrolimus | 111 (94.1) | 57 (98.3) | 54 (90.0) |  |
| Number of medication other than IS, median (IQR) | 3.0 (2.0–5.0) | 3.0 (2.0–4.0) | 3.0 (1.5–5.0) | 0.45 |
| Previous acute rejection, *n* (%) |  |  |  | 0.28 |
| None | 84 (71.8) | 39 (67.2) | 45 (76.3) |  |
| 1 | 25 (21.4) | 16 (27.6) | 9 (15.3) |  |
| 2 | 6 (5.1) | 2 (3.4) | 4 (6.8) |  |
| ≥ 3 | 2 (1.8) | 1 (1.7) | 1 (1.7) |  |
| Serious infection after transplantation, *n* (%) | 23 (19.5) | 13 (22.4) | 10 (16.7) | 0.58 |
| Systolic blood pressure (mmHg), mean±SD | 122.9 ± 10.7 | 121.5 ± 9.3 | 124.2 ± 11.8 | 0.17 |
| Serum creatinine (mg/dl), median (IQR) | 1.2 (1.0–1.4) | 1.2 (1.0–1.5) | 1.3 (1.0–1.3) | 0.38 |
| MDRD GFR (ml/min/1.73 m^2^), mean±SD | 65.4 ± 15.6 | 65.8 ± 16.7 | 65.1 ± 14.5 | 0.82 |
| 6 mo. intraindividual variability of CNI, mean±SD | 14.3 ± 8.0 | 14.7 ± 8.2 | 13.9 ± 7.9 | 0.60 |
| HADS anxiety score, median (IQR) | 5 (3–7) | 5 (3–7) | 4 (3–7) | 0.47 |
| HADS depression score, median (IQR) | 6 (4–8) | 6 (3–8) | 5.5 (4–8) | 0.83 |
| BFI-10 neuroticism score, median (IQR) | 2.8 (2.0–3.5) | 3.0 (2.0–3.5) | 2.5 (2.0–3.2) | 0.52 |
| BFI-10 openness score, median (IQR) | 3.5 (3.0–4.0) | 3.2 (2.5–4.0) | 3.5 (3.0–4.0) | 0.87 |
| BFI-10 extraversion score, median (IQR) | 3.0 (2.8–3.3) | 3.0 (2.8–3.3) | 2.9 (2.9–3.3) | 0.14 |
| BFI-10 agreeableness score, median (IQR) | 3.5 (3.0–4.0) | 3.5 (3.0–4.0) | 3.5 (3.0–4.0) | 0.69 |
| BFI-10 conscientiousness score, median (IQR) | 3.5 (3.0–4.0) | 3.5 (2.5–4.0) | 3.5 (3.0–4.0) | 0.49 |

SD, standard deviation; BMI, body mass index; ESRD, end stage renal disease; IgA, immunoglobulin A; ADPKD, autosomal dominant polycystic kidney disease; FSGS, focal segmental glomerulosclerosis; SLE, systemic lupus erythematosus; HSN, Henoch Schönlein nephritis; IS, immunosuppressant; IQR, interquartile range; MDRD GFR, glomerular filtration rate by Modification in Diet in Renal Disease study equation; CNI, calcineurin inhibitor; HADS, Hospital Anxiety and Depression Scale; BFI-10, 10-item Big Five Inventory; BAASIS, Basel Assessment of Adherence to Immunosuppressive Medication Scale; VAS, Visual Analog Scale.
