# Supplementary material for: Mobile medication manager application to improve adherence with immunosuppressive therapy in renal transplant recipients: A randomized controlled trial
Source: PLoS One. 2019 Nov 5;14(11):e0224595. doi: 10.1371/journal.pone.0224595 (PMC6830819; doi:10.1371/journal.pone.0224595)
Supplement: S3 Table — (DOCX) [file pone.0224595.s006.docx]

**S3 Table. Clinical factors associated with baseline self-rated nonadherence by BAASIS**

|  | **Univariate analysis** | | | **Multivariate analysis^a^** | |
| --- | --- | --- | --- | --- | --- |
|  | **Adherent**  **(*n*=63)** | **Nonadherent**  **(*n*=73)** | **P-value** | **OR**  **(95% CI)** | **P-value** |
| Age (years), median (IQR) | 41.0 (35.0–53.5) | 44.0 (31.0–53.0) | 0.81 |  |  |
| BMI (kg/m^2^), mean ± SD | 22.3 ± 3.3 | 22.1 ± 2.9 | 0.74 |  |  |
| Male sex, *n* (%) | 39 (61.9) | 49 (67.1) | 0.65 |  |  |
| Education level, *n* (%) |  |  | 0.93 |  |  |
| Less than middle school | 4 ( 6.3) | 3 ( 4.1) |  |  |  |
| Middle school | 9 (14.3) | 10 (13.7) |  |  |  |
| Highschool | 21 (33.3) | 27 (37.0) |  |  |  |
| University | 29 (46.0) | 33 (45.2) |  |  |  |
| **Occupation : employed or student, *n* (%)** | **36 (57.1)** | **53 (72.6)** | **0.09** |  |  |
| **Smoking, *n* (%)** | **0** | **5 ( 6.8)** | **0.10** | **–** | 1.00 |
| Dialysis before transplantation, *n* (%) | 51 (81.0) | 63 (86.3) | 0.54 |  |  |
| Dialysis duration (months), median (IQR) | 14.6 (2.5–63.2) | 36.0 (5.5–66.6) | 0.24 |  |  |
| **≥ 2yr post-transplantation, *n* (%)** | **23 (36.5)** | **46 (63.0)** | **0.004** | **3.07**  **(1.44–6.58)** | **0.004** |
| **Donor type, *n* (%)** |  |  | **0.04** |  |  |
| **- First degree relative or spouse** | **29 (46.0)** | **19 (26.0)** |  |  |  |
| **- Other living donor** | **10 (15.9)** | **21 (28.8)** |  | **5.26**  **(1.78–15.54)** | **0.003** |
| **- Deceased donor** | **24 (38.1)** | **33 (45.2)** |  | **2.97**  **(1.23–7.17)** | **0.02** |
| Second transplantation, *n* (%) | 5 (7.9) | 3 ( 4.1) | 0.56 |  |  |
| Number of IS – 2 (versus 3) , *n* (%) | 10 (15.9) | 13 (18.6) | 0.94 |  |  |
| Tacrolimus as calcineurin inhibitor, *n* (%) | 60 (95.2) | 68 (93.2) | 0.88 |  |  |
| Number of comedication, median (IQR) | 3.0 (2.0–5.0) | 3.0 (2.0–5.0) | 0.68 |  |  |
| Previous acute rejection, *n* (%) | 19 (30.2) | 25 (34.2) | 0.75 |  |  |
| Pevious serious infection, *n* (%) | 14 (22.2) | 11 (15.1) | 0.39 |  |  |
| MDRD GFR, median (IQR) | 61.4 (53.7–71.5) | 64.3 (52.7–74.3) | 0.54 |  |  |
| 6 month IIV of CNI, median (IQR) | 13.5 (8.5–19.0) | 11.9 (8.6–18.0) | 0.54 |  |  |
| **HADS anxiety score ≥ 8, *n* (%)** | **8 (12.7)** | **19 (26.0)** | **0.08** |  |  |
| HADS depression score ≥ 8, *n* (%) | 15 (23.8) | 26 (35.6) | 0.19 |  |  |
| BFI-10 neuroticism score, median (IQR) | 2.5 (2.0–3.5) | 3.0 (2.0–3.5) | 0.83 |  |  |
| BFI-10 openness score, median (IQR) | 3.5 (2.8–4.0) | 3.5 (3.0–4.0) | 0.25 |  |  |
| BFI-10 extraversion score, median (IQR) | 3.0 (2.6–3.2) | 3.0 (2.5–3.2) | 0.86 |  |  |
| BFI-10 agreeableness score, median (IQR) | 3.5 (3.0–4.5) | 3.5 (3.0–4.5) | 0.11 |  |  |
| **BFI-10 conscientiousness score, median (IQR)** | **3.5 (3.0–4.5)** | **3.5 (2.5–4.0)** | **0.06** | **0.57**  **(0.36–0.88)** | **0.01** |

IQR, interquartile range; SD, standard deviation; BMI, body mass index; IS, immunosuppressant; MDRD GFR, glomerular filtration rate by Modification in Diet in Renal Disease study equation; CNI, calcineurin inhibitor; IIV, intraindividual variability; HADS, Hospital Anxiety and Depression Scale; BFI-10, 10-item Big Five Inventory; BAASIS, Basel Assessment of Adherence to Immunosuppressive Medication Scale; VAS, Visual Analog Scale.

^a^ Multivariate logistic regression analysis including factors with P<0.1 in the univariate analysis
