# Supplementary material for: Mobile medication manager application to improve adherence with immunosuppressive therapy in renal transplant recipients: A randomized controlled trial
Source: PLoS One. 2019 Nov 5;14(11):e0224595. doi: 10.1371/journal.pone.0224595 (PMC6830819; doi:10.1371/journal.pone.0224595)
Supplement: S4 Table — (DOCX) [file pone.0224595.s007.docx]

**S4 Table. Clinical factors associated with baseline self rated nonadherence by VAS**

|  | **Univariate analysis** | | | **Multivariate analysis** ^a^ | |
| --- | --- | --- | --- | --- | --- |
|  | **Adherent**  **(*n*=66)** | **Nonadherent**  **(*n*=70)** | **P-value** | **OR**  **(95% CI)** | **P-value** |
| **Age (years), median (IQR)** | **46.0 (35.0–56.0)** | **43.0 (29.0–51.0)** | **0.04** |  |  |
| BMI (kg/m^2^), mean ± SD | 22.2 ± 3.0 | 22.2 ± 3.3 | 0.96 |  |  |
| **Male sex, *n* (%)** | **37 (56.1)** | **51 (72.9)** | **0.06** | **2.32**  **(1.07 – 5.03)** | **0.03** |
| Education level, *n* (%) |  |  | 0.45 |  |  |
| Less than middle school | 5 ( 7.6) | 2 ( 2.9) |  |  |  |
| Middle school | 9 (13.6) | 10 (14.3) |  |  |  |
| Highschool | 20 (30.3) | 28 (40.0) |  |  |  |
| University | 32 (48.5) | 30 (42.9) |  |  |  |
| **Occupation : employed or student**, *n* (%) | **37 (56.1)** | **52 (74.3)** | **0.04** |  |  |
| **Smoking**, *n* (%) | **0** | **5 ( 7.1)** | **0.08** | – | 1.00 |
| Dialysis before TPL, *n* (%) | 54 (81.8) | 60 (85.7) | 0.70 |  |  |
| Dialysis duration (months), median (IQR) | 21.7 (1.4–65.6) | 30.6 (5.5–72.0) | 0.36 |  |  |
| ≥ 2yr posttransplantation, *n* (%) | 29 (43.9) | 40 (57.1) | 0.17 |  |  |
| Donor type, *n* (%) |  |  | 0.90 |  |  |
| - first degree relative or spouse | 13 (19.7) | 15 (21.4) |  |  |  |
| - Other living donor | 24 (36.4) | 27 (38.6) |  |  |  |
| - Deceased donor | 29 (43.9) | 28 (40.0) |  |  |  |
| 2nd transplantation, *n* (%) | 6 ( 9.1) | 2 ( 2.9) | 0.24 |  |  |
| Number of IS – 2 (versus 3) , *n* (%) | 10 (15.2) | 13 (18.6) | 0.76 |  |  |
| Tacrolimus as CNI, *n* (%) | 62 (93.9) | 66 (94.3) | 1.00 |  |  |
| No. of comedication, median (IQR) | 3.0 (2.0–5.0) | 3.0 (2.0–5.0) | 0.64 |  |  |
| **Previous acute rejection, *n* (%)** | **16 (24.2)** | **28 (40.0)** | **0.08** |  |  |
| Pevious serious infection, *n* (%) | 11 (16.7) | 14 (20.0) | 0.78 |  |  |
| MDRD GFR, median (IQR) | 63.2 (53.7–76.0) | 61.7 (52.7–72.2) | 0.51 |  |  |
| 6 mo. IIV of CNI, median (IQR) | 13.8 (9.7–21.1) | 11.7 (7.1–18.1) | 0.24 |  |  |
| **HADS anxiety score ≥ 8, *n* (%)** | **6 ( 9.1)** | **21 (30.0)** | **0.005** |  |  |
| **HADS depression score ≥ 8, *n* (%)** | **13 (19.7)** | **28 (40.0)** | **0.02** |  |  |
| BFI-10 neuroticism score, median (IQR) | 2.5 (2.0–3.0) | 3.0 (2.0–3.5) | 0.45 |  |  |
| BFI-10 openness score, median (IQR) | 3.5 (3.0–4.0) | 3.5 (3.0–4.0) | 0.83 |  |  |
| BFI-10 extraversion score, median (IQR) | 3.0 (2.8–3.2) | 3.0 (2.5–3.2) | 0.85 |  |  |
| BFI-10 agreeableness score, median (IQR) | 3.5 (3.0–4.0) | 3.5 (3.0–4.0) | 0.53 |  |  |
| **BFI-10 conscientiousness score, median (IQR)** | **3.5 (3.5–4.5)** | **3.5 (2.5–4.0)** | **0.001** | **0.45**  **(0.29 – 0.71)** | **<0.001** |

IQR, interquartile range; SD, standard deviation; BMI, body mass index; IS, immunosuppressant; MDRD GFR, glomerular filtration rate by Modification in Diet in Renal Disease study equation; CNI, calcineurin inhibitor; IIV, intraindividual variability; HADS, Hospital Anxiety and Depression Scale; BFI-10, 10-item Big Five Inventory; BAASIS, Basel Assessment of Adherence to Immunosuppressive Medication Scale; VAS, Visual Analog Scale.

^a^ Multivariate logistic regression analysis including factors with P<0.1 in the univariate analysis
