# Supplementary material for: Mobile medication manager application to improve adherence with immunosuppressive therapy in renal transplant recipients: A randomized controlled trial
Source: PLoS One. 2019 Nov 5;14(11):e0224595. doi: 10.1371/journal.pone.0224595 (PMC6830819; doi:10.1371/journal.pone.0224595)
Supplement: S5 Table — GEE analysis for the interventional effect on (A) dosing and (B) timing by MEMS. (DOCX) [file pone.0224595.s008.docx]

**S5 Table. GEE analysis for the interventional effect on (A) correct dosing and (B) correct timing over time**

(A)

|  | Dosing | | |
| --- | --- | --- | --- |
|  | Wald's chi square | Degree of freedom | p |
| (Adjusted model) | 98.021 | 1 | 0.000 |
| Study group | 0.410 | 1 | 0.522 |
| Time (EM days) | 14.487 | 1 | 0.000 |
| Study group x time | 0.098 | 1 | 0.755 |

(B)

|  | Timing | | |
| --- | --- | --- | --- |
|  | Wald's chi square | Degree of freedom | p |
| (Adjusted model) | 117.542 | 1 | 0.000 |
| Study group | 0.086 | 1 | 0.770 |
| Time (EM days) | 16.540 | 1 | 0.000 |
| Study group x time | 0.094 | 1 | 0.760 |
