# Supplementary material for: Mobile medication manager application to improve adherence with immunosuppressive therapy in renal transplant recipients: A randomized controlled trial
Source: PLoS One. 2019 Nov 5;14(11):e0224595. doi: 10.1371/journal.pone.0224595 (PMC6830819; doi:10.1371/journal.pone.0224595)
Supplement: S6 Table — (DOCX) [file pone.0224595.s009.docx]

**S6 Table. Secondary outcomes**

|  | **Total (N=136)** | **Control group (N=66)** | **Mobile group (N=70)** | **Effect estimate**  **(Odd ratio (95% CI),**  **Cohen’s d, or η^2^)** | **P-value** |
| --- | --- | --- | --- | --- | --- |
| GFR at 180 day, mean ± SD | 63.8 ± 15.3 | 62.3 ± 15.3 | 65.4 ± 15.4 | 0.20 | 0.311 |
| ∆GFR, mean ± SD | -1.8 ± 8.0 | -2.6 ± 7.6 | -1.0 ± 8.4 | 0.20 | 0.321 |
| Acute rejection, n (%) | 3 (2.2%) | 1 (1.5%) | 2 (2.9%) | 1.91 (0.29–7.33) | 1.000 |
| IIV during the study period, median (IQR) | 13.1 (8.4**–**18.6) | 13.3 (8.4**–**18.7) | 12.5 (8.2**–**18.4) | 0.0001 | 0.906 |

Effect estimate for outcomes of the mobile group are presented in reference to the control group. Effect estimate is presented with and the odds ratio with 95% confidence interval for the chi-squared (χ^2^) test or Fisher’s exact test, Cohen’s d for independent sample t-tests, and η^2^ ($=\frac{Z^{2}}{\sqrt{n}}$) for the Mann–Whitney U test.

eGFR, estimated glomerular filtration rate; ∆ eGFR, difference in eGFR (GFR at visit 3 – GFR at visit 0); IIV, intraindividual variability
