# Supplementary material for: Mobile medication manager application to improve adherence with immunosuppressive therapy in renal transplant recipients: A randomized controlled trial
Source: PLoS One. 2019 Nov 5;14(11):e0224595. doi: 10.1371/journal.pone.0224595 (PMC6830819; doi:10.1371/journal.pone.0224595)
Supplement: S7 Table — (DOCX) [file pone.0224595.s010.docx]

**S7 Table. Adherence of patients according to their app usage**

|  | **Total mobile group (*n* = 70)** | **App use stopped before 28 days^b^**  **(*n* =39)** | **Continued**  **app use ≥28 days^b^ (***n* ***=31*)** | **P-value** |
| --- | --- | --- | --- | --- |
| ***Baseline*** |  |  |  |  |
| self-rated nonadherence by BAASIS, *n* (%) | 36 (51.4%) | 19 (48.7%) | 17 (54.8%) | 0.79 |
| self-rated nonadherence by VAS, *n* (%) | 32 (45.7%) | 18 (46.2%) | 14 (45.2%) | 1.00 |
| ***Initial visit (0mo) – Visit 1 (1mo)*** |  |  |  |  |
| Taking adherence (%), median (IQR)^a^ | 97.6 (82.9 –100) | 92.9 (71.4 –100) | 98.2 (96.4 –100) | 0.02 |
| Dosing adherence (%), median (IQR) ^a^ | 94.0 (68.0 –100) | 82.1 (51.2 –100) | 95.2 (90.5–100) | 0.04 |
| Timing adherence (%), median (IQR) ^a^ | 95.0 (61.1 –100) | 75.0 (51.8 –100) | 96.3 (92.5 –100) | 0.04 |
| Drug holiday (day), median (IQR) ^a^ | 0 (0 – 0) | 0 (0 – 1) | 0 (0– 0) | 0.07 |
| Overall nonadherence rate based on EM algorithm, *n* (%)^a^ | 32 (53.3%) | 20 (64.5%) | 12 (41.4%) | 0.12 |
| Self-rated nonadherence by BAASIS, *n* (%) | 16 (24.6%) | 11 (32.4%) | 5 (16.1%) | 0.22 |
| Self-rated nonadherence by VAS, *n* (%) | 29 (44.6%) | 18 (52.9%) | 11 (35.5%) | 0.24 |

^a^ EM prevalences were calculated in 60 patients (31 patients who stopped using app before 28 days and 29 patients who continued to use app beyond 28 days whose EM values were available

**^b^** App usage rate was assessed based on the time log data of the reminder function of the app
