# Supplementary material for: Mobile medication manager application to improve adherence with immunosuppressive therapy in renal transplant recipients: A randomized controlled trial
Source: PLoS One. 2019 Nov 5;14(11):e0224595. doi: 10.1371/journal.pone.0224595 (PMC6830819; doi:10.1371/journal.pone.0224595)
Supplement: S8 Table — (DOCX) [file pone.0224595.s011.docx]

**S8 Table. Baseline characteristics of the patients according to their app usage**

|  | **Total mobile group n = 70** | **App use stopped before 28days ^a^**  **(n=39)** | **Continued**  **app use ≥28days ^a^ (n=31)** | **P-value** |
| --- | --- | --- | --- | --- |
| ***Sociodemographics*** |  |  |  |  |
| Age (years), median (IQR) | 45.0 (35.0–54.0) | 47.0 (37.0–53.0) | 43.0 (35.0–54.0) | 0.84 |
| BMI (kg/m^2^), mean ± SD | 22.5 ± 3.4 | 22.9 ± 3.6 | 21.9 ± 3.2 | 0.21 |
| Male | 43 (61.4%) | 25 (64.1%) | 18 (58.1%) | 0.79 |
| Education level |  |  |  | 0.42 |
| Less than middle school | 2 ( 2.9%) | 0 ( 0.0%) | 2 ( 6.5%) |  |
| Middle school | 11 (15.7%) | 7 (17.9%) | 4 (12.9%) |  |
| Highschool | 27 (38.6%) | 15 (38.5%) | 12 (38.7%) |  |
| University | 30 (42.9%) | 17 (43.6%) | 13 (41.9%) |  |
| Occupation |  |  |  | 0.27 |
| Full time | 37 (52.9%) | 20 (51.3%) | 17 (54.8%) |  |
| Part time | 4 ( 5.7%) | 1 ( 2.6%) | 3 ( 9.7%) |  |
| Student | 5 ( 7.1%) | 4 (10.3%) | 1 ( 3.2%) |  |
| Housewife | 14 (20.0%) | 10 (25.6%) | 4 (12.9%) |  |
| Unemployed | 10 (14.3%) | 4 (10.3%) | 6 (19.4%) |  |
| Smoking |  |  |  | 0.44 |
| Current smoker | 1 ( 1.4%) | 1 ( 2.6%) | 0 ( 0.0%) |  |
| Previous smoker | 1 ( 1.4%) | 1 ( 2.6%) | 0 ( 0.0%) |  |
| Non smoker | 68 (97.1%) | 37 (94.9%) | 31 (100.0%) |  |
| ***Clinical characteristics*** |  |  |  |  |
| Causes of ESRD |  |  |  | 0.80 |
| IgA nephropathy | 13 (18.6%) | 7 (17.9%) | 6 (19.4%) |  |
| Glomerulonephritis | 8 (11.4%) | 5 (12.8%) | 3 ( 9.7%) |  |
| ADPKD | 9 (12.9%) | 5 (12.8%) | 4 (12.9%) |  |
| Hypertension | 5 ( 7.1%) | 4 (10.3%) | 1 ( 3.2%) |  |
| Diabetes | 4 ( 5.7%) | 3 ( 7.7%) | 1 ( 3.2%) |  |
| FSGS | 5 ( 7.1%) | 2 ( 5.1%) | 3 ( 9.7%) |  |
| Vesicoureteral reflux | 3 ( 4.3%) | 2 ( 5.1%) | 1 ( 3.2%) |  |
| SLE | 1 ( 1.4%) | 1 ( 2.6%) | 0 ( 0.0%) |  |
| HSN | 2 ( 2.9%) | 0 ( 0.0%) | 2 ( 6.5%) |  |
| unknown | 18 (25.7%) | 9 (23.1%) | 9 (29.0%) |  |
| others | 2 ( 2.9%) | 1 ( 2.6%) | 1 ( 3.2%) |  |
| Dialysis before TPL | 56 (80.0%) | 32 (82.1%) | 24 (77.4%) | 0.86 |
| Dialysis duration, median months (IQR) | 29.0 ( 2.8–72.0) | 34.3 ( 5.2–82.8) | 9.2 ( 2.9–53.9) | 0.25 |
| Months since TPL, median (IQR) | 27.2 (14.2–57.4) | 24.8 (13.5–56.9) | 29.7 (18.6–52.5) | 0.47 |
| Donor type |  |  |  |  |
| Living donor |  |  |  | 0.65 |
| - first degree related | 14 (20.0%) | 9 (23.1%) | 5 (16.1%) |  |
| - spouse | 11 (15.7%) | 7 (17.9%) | 4 (12.9%) |  |
| - other living donor | 16 (22.9%) | 7 (17.9%) | 9 (29.0%) |  |
| Deceased donor | 29 (41.4%) | 16 (41.0%) | 13 (41.9%) |  |
| Number of transplantation |  |  |  | 1.00 |
| First | 67 (95.7%) | 37 (94.9%) | 30 (96.8%) |  |
| Second | 3 ( 4.3%) | 2 ( 5.1%) | 1 ( 3.2%) |  |
| Number of IS |  |  |  | 1.00 |
| 2 | 9 (12.9%) | 5 (12.8%) | 4 (12.9%) |  |
| 3 | 61 (87.1%) | 34 (87.2%) | 27 (87.1%) |  |
| Number of medication other than IS, median (IQR) | 3.5 ( 2.0– 5.0) | 4.0 ( 2.0– 5.0) | 3.0 ( 1.5– 5.0) | 0.29 |
| Previous acute rejection |  |  |  | 0.32 |
| None | 51 (73.9%) | 25 (65.8%) | 26 (83.9%) |  |
| 1 | 12 (17.4%) | 8 (21.1%) | 4 (12.9%) |  |
| 2 | 5 ( 7.2%) | 4 (10.5%) | 1 ( 3.2%) |  |
| ≥ 3 | 1 ( 1.4%) | 1 ( 2.6%) | 0 ( 0.0%) |  |
| Serious infection after transplantation | 11 (15.7%) | 6 (15.4%) | 5 (16.1%) | 1.00 |
| Systolic blood pressure, mean ± SD | 123.9 ± 11.2 | 123.3 ± 11.3 | 124.6 ± 11.3 | 0.63 |
| Serum creatinine, mean ± SD | 1.2 ± 0.3 | 1.2 ± 0.3 | 1.2 ± 0.3 | 0.86 |
| MDRD GFR, mean ± SD | 65.1 ± 14.2 | 65.4 ± 12.2 | 64.8 ± 16.6 | 0.87 |
| 6 mo. IIV of CNI, median (IQR) | 11.6 ( 8.2–17.8) | 12.4 ( 8.2–17.8) | 10.9 ( 8.4–18.8) | 0.97 |
| HADS anxiety score ≥ 8 | 13 (18.6%) | 8 (20.5%) | 5 (16.1%) | 0.87 |
| HADS depression score ≥ 8 | 22 (31.4%) | 12 (30.8%) | 10 (32.3%) | 1.00 |
| BFI-10 neuroticism score, median (IQR) | 2.8 (2.0–3.5) | 3.0 (2.5–3.5) | 2.5 (2.0–3.0) | 0.11 |
| BFI-10 openness score, median (IQR) | 3.5 (3.0–4.0) | 3.5 (3.0–4.0) | 3.5 (3.0–4.0) | 0.92 |
| BFI-10 extraversion score, median (IQR) | 3.0 (2.5–3.3) | 3.0 (2.5–3.3) | 2.8 (2.5–3.3) | 0.90 |
| BFI-10 agreeableness score, median (IQR) | 3.5 (3.0–4.0) | 3.5 (3.0–4.0) | 3.5 (3.0–4.0) | 0.78 |
| BFI-10 conscientiousness score, median (IQR) | 3.5 ( 3.0**–** 4.0) | 3.5 ( 3.0– 4.0) | 3.5 ( 3.0– 4.0) | 0.97 |

Values are *n* (%) unless specified otherwise.

IQR, interquartile range; SD, standard deviation; BMI, body mass index; TPL, transplantation; ESRD, end stage renal disease; IgA, immunoglobulin A; ADPKD, autosomal dominant polycystic kidney disease; FSGS, focal segmental glomerulosclerosis; SLE, systemic lupus erythematosus; HSN, Henoch Schönlein nephritis; IS, immunosuppressant; MDRD GFR, glomerular filtration rate by Modification in Diet in Renal Disease study equation; CNI, calcineurin inhibitor; IIV, intraindividual variability; HADS, Hospital Anxiety and Depression Scale; BFI-10, 10-item Big Five Inventory; BAASIS, Basel Assessment of Adherence to Immunosuppressive Medication Scale; VAS, Visual Analog Scale.

**^a^** App usage rate was assessed based on the time log data of the reminder function of the app
